# Proteomic Characterization of Collagen-Based Animal Glues for Restoration

**DOI:** 10.1021/acs.jproteome.2c00232

**Published:** 2022-08-15

**Authors:** Georgia Ntasi, Sara Sbriglia, Rossana Pitocchi, Roberto Vinciguerra, Chiara Melchiorre, Laura Dello Ioio, Giancarlo Fatigati, Emanuele Crisci, Ilaria Bonaduce, Andrea Carpentieri, Gennaro Marino, Leila Birolo

**Affiliations:** † Department of Chemical Sciences, University of Naples Federico II, Complesso Universitario di Monte S. Angelo, Via Cinthia 21, 80126 Naples, Italy; ‡ Dello Ioio Restauri, Vico Equense, 80069 Naples, Italy; § Department of Humanities, University Suor Orsola Benincasa, via Santa Caterina da Siena 37, 80132, Naples, Italy; ⊥ Department of Chemistry and Industrial Chemistry, University of Pisa, via Risorgimento 35, 56126 Pisa, Italy; ¶ Task Force “Metodologie Analitiche per la Salvaguardia dei Beni Culturali”, University of Naples Federico II, 80138 Naples, Italy

**Keywords:** animal glue, collagen, LC-MSMS, GC-MS, protein degradation, protein aging, deamidation, protein modification, proteomics

## Abstract

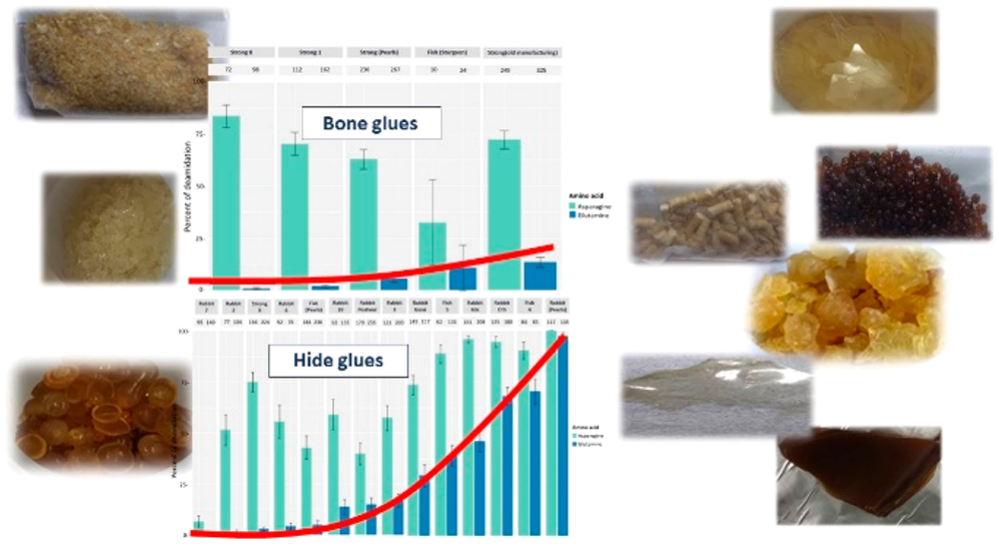

Animal glues are widely used in restoration as adhesives, binders, and consolidants for organic and inorganic materials. Their variable performances are intrinsically linked to the adhesive properties of collagen, which determine the chemical, physical, and mechanical properties of the glue. We have molecularly characterized the protein components of a range of homemade and commercial glues using mass spectrometry techniques. A shotgun proteomic analysis provided animal origin, even when blended, and allowed us to distinguish between hide and bone glue on the basis of the presence of collagen type III, which is abundant in connective skin/leather tissues and poorly synthetized in bones. Furthermore, chemical modifications, a consequence of the preparation protocols from the original animal tissue, were thoroughly evaluated. Deamidation, methionine oxidation, and backbone cleavage have been analyzed as major collagen modifications, demonstrating their variability among different glues and showing that, on average, bone glues are less deamidated than hide glues, but more fragmented, and mixed-collagen glues are overall less deamidated than pure glues. We believe that these data may be of general analytical interest in the characterization of collagen-based materials and may help restorers in the selection of the most appropriate materials to be used in conservation treatments.

## Introduction

Animal glues are widely used in restoration, serving as adhesives, binders, coatings, and consolidants for organic and inorganic materials.^1,2^ The term animal glue usually refers to an adhesive prepared from vertebrate connective tissues, namely, bones, skin/hide, or sinew. Upon treatment with acids or alkalis in hot water, the otherwise insoluble collagen, the main constituent protein of all these tissues, becomes soluble. The first archeological evidence of collagen-based coatings was identified in baskets from the Nahal Hemal cave (Israel, ca. 8200–7300 BC).^3^ The earliest finding of animal-based adhesives in Europe dates back to the fourth millennium BC, when farmers in the Zurich area performed rudimentary chemical extractions to produce hide glue, most likely from the skins and other collagen-rich connective tissues of domestic cattle and ovicaprids.^4^

A simple procedure for making animal glue was reported in 2000 BC, while the first commercial glue factory was started in the 1700s in Holland, where animal glue was made from hides, and the first patent was issued for a fish glue in Britain about 50 years later.^2^ Later on, there was a flourish of patents for glue recipes made from bones and skins of slaughtered animals, fish, starch, and milk.^5^ Leather glues come from tannery waste, while bone glues are obviously made from animal bones. Fish glues are nonedible byproducts from fisheries such as skins, bones, cartilages, and swim bladders.

All animal glues are made from collagen. Collagen in its natural state is a triple helix protein and has the distinctive Gly–X–Y repetitive sequence and a unique high content of Pro and Hyp, making it easily recognizable in the protein universe. It is naturally insoluble in water, and it must be processed into soluble gelatin to be used as animal glue.

The performance of the glue depends on the original source of collagen but is also strongly influenced by the extraction and preparation procedures, since both the specific sequence and the processing of the starting material affect the resistance of the polypeptide chains to hydrolysis and the (partial) denaturation of the triple helix that occurs upon heating in the gelatinization process.^5^

These factors have a significant influence on glue properties, in terms of viscosity, strength, and overall mechanical behavior.^6^ The conservators’ choice of commercial glue to use is primarily based on their empirical experience and often lacks a scientific characterization that could help them in making a properly informed decision.

In this regard, this paper aims to provide a diagnostic protocol that can be used to molecularly characterize commercial animal glues from different sources. With a variety of different animal glues on the market, such as hide and bone glues, fish glues, isinglass, and gelatin, their individual molecular properties must be well understood to link them to specific products. However, while the identification of collagen-based glues in artworks and their discrimination from other types of protein binders, such as milk and egg, for instance, can be easily performed based on the Gly, Pro, and Hyp peculiar high content,^7^ unambiguous species determination is made difficult by the repetitiveness of the collagen sequence pattern and, more importantly, by the extreme sequence conservation. In addition, species identification becomes extremely challenging with samples containing multiple glues derived from different animals. It is also well-known that some suppliers provide rabbit glue mixed with bovine glue to alter its properties.^6^ In this regard, proteomic analysis is the method of choice.

Proteomics is gaining momentum in the field of diagnostic tools for cultural heritage. Besides being of interest to art historians and archeologists in the characterization of materials and the state of conservation of artworks or archeological remains, it can also be useful to conservators during the selection of the most suitable materials to be used. Due to the high accuracy and sensitivity of mass spectrometry, proteomics has already been widely used for collagen analysis to discriminate bone fragments of different animal species^8−10^ and to identify protein binders in paintings^11,12^ and gilt samples.^13^

Moreover, proteomic analysis is extremely powerful in addressing the problem of characterizing the changes that occur during collagen degradation in animal glue because, as clearly stated by Schellmann,^6^ during the extraction and preparation procedure, collagen chemistry can be significantly altered. Molecular weight distribution is certainly affected by preparation protocols, as is pI, which can be altered by extensive deamidation, one of the most common chemical modifications in damaged proteins.^14,15^ What happens to collagen during glue preparation can therefore significantly affect the glue properties. Manufacturers tend to keep their recipes secret, and collagen-derived performance is therefore not easily predictable based on the label alone. Using a combination of mass spectrometric techniques (GC-MS, LC-MS/MS, and Py-GC-MS) and denaturing electrophoresis, we have provided extensive molecular characterization of a set of glue samples to give a broad picture in terms of constituent proteins and their modifications.

## Experimental Section

### Animal Glue Samples

A set of 19 animal glues provided by the restoration workshop of the University Suor Orsola Benincasa in Naples, and by Museo Nacional del Prado in Madrid, have been analyzed and characterized. The label of the samples along with a picture, their reported source, and new classification in **H**ide and **B**one, **P**ure and **M**ixed, and **F**ish **M**ixed on the basis of proteomic analysis herein carried out are reported in Table S1.

### Gel Electrophoresis under Denaturing Conditions (SDS-PAGE)

Acid-soluble collagen (ASC) was extracted as reported in Hong et al 2017,^16^ with slight modifications. Each animal glue sample (10 mg) was dissolved in a solution of acetic acid 0.5 M (1:10 w/v) under continuous stirring for 24 h. The solution was centrifuged at 10000*g* for 15 min at 4 °C. The supernatant, which consists of acid-soluble collagen (ASC), was collected and extensively dialyzed (10 kDa cut off) in a solution of ammonium bicarbonate 1.26 M at 4 °C for 24 h. pH was measured, verifying neutralization. Subsequently, 10 μL of each acid soluble collagen (ACS) fraction were diluted with 8 μL of sample buffer (65.8 mM Tris-HCI, pH 6.8, 2.1% SDS, 26.3% (w/v) glycerol, 0.01% bromophenol blue) containing 0.1 M DTT and heat denatured at 100 °C for 10 min. The samples were loaded onto a monodimensional SDS-PAGE (7%). To visualize the protein bands, gel was stained with Coomassie brilliant blue. Gel was then scanned with ChemiDoc MP imaging system (Bio-Rad).

### Protein Extraction and Digestion

Samples were prepared as reported in ref (17). Briefly, 1–2 mg of each pellet was resuspended in 10 μL of 6 M urea. Samples were incubated for 20 min at room temperature and then for 30 min in the sonicator. Samples were then 6-fold diluted with 10 mM ammonium bicarbonate at pH 7.5, and enzymatic digestion was carried out by the addition of 1 μg of trypsin at 37 °C for 16 h. The supernatants were then recovered by centrifugation and filtered on 0.22 μm PVDF membrane (Millipore), and peptides were desalted and concentrated on in-house made C18 extraction stage tips as described by Cappellini et al.^32^ Peptides were eluted with 20 μL of 50% acetonitrile and 0.1% formic acid in Milli-Q water and analyzed by LC-MS/MS.

### LC-MS/MS

Samples were analyzed on a 6520 Accurate-Mass Q-Tof LC/MS System (Agilent Technologies, Palo Alto, CA, U.S.A.) equipped with a 1200 HPLC System and a chip cube (Agilent Technologies) as reported in ref (17). Samples were fractionated on a C18 reverse-phase capillary column (Agilent Technologies) at a flow rate of 400 nL/min, with a linear gradient of eluent B (0.1% formic acid in 95% acetonitrile) in A (0.1% formic acid in 2% aceto-nitrile) from 3% to 80% in 50 min. Peptide analysis was performed using data-dependent acquisition (DDA) of one MS scan (mass range from 300 to 2000 *m*/*z*) followed by MS/MS scans of the three most abundant ions in each MS scan. MS/MS spectra were measured automatically when the MS signal surpassed the thresh-old of 50 000 counts. Double and triple charged ions were preferably isolated and fragmented.

Alternatively, peptide fractionation was performed on LTQ Orbitrap XL Hybrid Ion Trap-Orbitrap MS System (Thermo Scientific, Bremen, Germany) on a C18 capillary reverse-phase column (200 mm, 75 μm, 5 μm) at 250 nl min^–1^ flow rate, using a step gradient of eluent B (0.2% formic acid, 95% acetonitrile LC-MS grade) in eluent A (0.2% formic acid in 2% acetonitrile) from 5 to 50% over 80 min and to 80% over 5 min. Mass spectrometric analyses were performed using data-dependent acquisition (DDA) mode over the 400 to 1800 mz^–1^ range, at a resolution of 60 000, and the automatic gain control (AGC) target was set to 1 × 10^6^, followed by acquisition in MS/MS of the five most abundant ions. For the MS/MS scans, the resolution was set to 15 000, the AGC target was set to 1 × 10^5^, the precursor isolation width was 2 Da, and the maximum injection time was set to 500 ms. The CID normalized collision energy was 35%; AGC target was set to 1 × 10^5^. Data were acquired by Xcalibur software (Thermo Fisher Scientific).

### Protein Identification and Semiquantitative Evaluation of Chemical Modifications

MS/MS spectra were transformed in Mascot Generic files (.mgf) format and routinely used to query the SwissProt database 2015_04 (548 208 sequences; 195 282 524 residues), with Chordata as the taxonomy restriction for protein identification. A licensed version of MASCOT software (www.matrixscience.com) version 2.4.0 was used. Standard parameters in the searches were trypsin as the enzyme (semitrypsin when searching for backbone cleavages); 3, as the allowed number of missed cleavages; 10 ppm MS tolerance and 0.6 Da MS/MS tolerance; and peptide charge from 2+ to 3+. In all the database searches, no fixed chemical modification was inserted, but possible oxidation of methionine residues, deamidation at asparagines and glutamines, and hydroxylation on lysine and proline were considered as variable modifications. To reduce the search space and recover more focused results, ultimate searches were carried out using a homemade database, which we named COLLE (60 sequences; 88 859 residues), that collects the sequences of collagen type I and III for all the common domesticates generally used for animal glues). Mass spectrometry data and the COLLE database have been deposited to Mendeley Data (https://data.mendeley.com/datasets/hbmc8yhf7y/2).

Semiquantitative evaluation of deamidation was carried out by MaxQuant software.^18^ Parameters common among all runs are as follows: tryptic search with up to two missed cleavages; minimum peptide length was set to 7; and no fixed modification was set, while oxidation of methionine, hydroxylation of proline and hydroxylation of lysine were set as variable modifications, with up to a maximum of 5 modifications per peptide. Protein identifications were supported by a false discovery rate (FDR) of 0.01 applied (same FDR for dependent peptides when applied) and manually filtered by at least 2 different nonoverlapping peptides above the 40 ion score threshold. Contaminant proteins were assessed using the contamination.fasta provided by MQ, which includes common laboratory contaminants (see MaxQuant Downloads -contaminants.fasta, can be found under http://www.coxdocs.org/doku.php?id=maxquant:start_downloads.htm, n.d). These protein hits were excluded from further analysis. After each run, the evidence file.txt of each animal glue was first cross validated for its peptides and proteins according to the protein identification that had already been performed with the use of Mascot. Afterward, the “evidence” files were used for the evaluation of deamidation (N, Q) level both in the total sample (global deamidation) and for the single polypeptide chain identified in each animal glue, with the public available software (https://github.com/dblyon/deamidation). The visualization of all deamidation plots was performed with the use of R studio.

Backbone cleavage evaluation was carried out by setting the same parameters as for standard protein identification as described above but “semitrypsin” as enzyme and an ion score cut off ≥ 25 for unmodified and modified peptides. The assessment of the occurrence of backbone cleavage was carried out by counting the PSMs (Peptide Spectrum Matches) in the single samples, focusing on Type I and Type III collagen chains only.

A site-specific evaluation of the deamidation (Asn, Gln) and oxidation (M) occurrence along the amino acidic sequence was performed by manually inspection of MS/MS data. The positions of Asn and Gln that were detected as unmodified were characterized as X; the positions that were found only deamidated as D; the position that were detected both as deamidated and unmodified as DX; and finally, the positions that were not detected at all, neither unmodified nor deamidated, as NF. Similarly, for the evaluation of oxidation, the detected oxidized positions were characterized as OX; those that have been detected as unmodified as X; and the nondetected as NF.

### GC-MS Analysis of Proteins

Samples (1–5 mg) were subjected to acidic hydrolysis in the vapor phase assisted by microwaves, at 160 °C with 6 M hydrochloric acid, power of 350W, for 35 min. At the end of the hydrolysis, samples were reconstituted with 300 μL of double-distilled water. Then 2 μL of the water solution of amino acids was then added with 5 μL of a standard solution of norleucine (internal standard, 73.77 ppm), dried under nitrogen flow, and subjected to silylation with *N*-methyl-*N*-(tert-butyldimethylsilyl)trifluoroacetamide (MTBSTFA). Analyses were carried out with a 6890N GC System Gas Chromatograph (Agilent Technologies, Palo Alto, CA, U.S.A.), coupled with a 5975 Mass Selective Detector (Agilent Technologies, Palo Alto, CA, U.S.A.) a single quadrupole mass spectrometer, equipped with a PTV injector. Samples were analyzed in duplicates. Quantitation was performed though calibration curves working in the SIM mode. Details of the operating conditions are reported in the literature.^19^

### Analytical Pyrolysis Coupled to GC-MS (Py-GC-MS)

Samples (ca. 100–200 μm) were subjected to flash pyrolysis at 550 °C for 0.2 min, and the interface temperature was 280 °C. The split/splitless injector was used at a 1:50 split ratio. The Pyrolyser was a Multi-Shot Pyrolyzer EGA/Py-3030D (Frontier Lab) coupled to a gas chromatograph 6890 coupled with a 5973 Mass Selective Detector Agilent Technologies (U.S.A.). Chromatographic and mass spectrometric conditions are reported in detail in the literature.^20^ To assess the simultaneous presence of other organic components, analytical pyrolysis was also carried out with *in situ* silylation. Samples (ca. 100–200 μm) were admixed to 2 μL of hexamethyldisilazane in the cup before the pyrolysis, carried out at 550 °C, Py-GC interface set at 280 °C, pyrolysis time of 0.2 min.

## Results and Discussion

### Protein Identification

#### Amino Acid Composition

The amino acid composition of the samples was determined by gas chromatography mass spectrometry. The samples were first hydrolyzed, then silylated, and finally analyzed by GC-MS. The quantitative determination of amino acids was performed by building calibration curves using standard solutions and evaluating daily recoveries. SIM mode was used for the quantitation. The quantified amino acids were: Ala, Gly, Val, Leu, Ile, Met, Ser, Pro, Phe, Asp, Glu, Hyp and Tyr. The results are listed in Table S2 where, for the sake of homogeneity, samples are named following classification upon proteomic analysis results

Data clearly show that the amino acid composition of the different samples is extremely similar to one another, as expected from samples based on collagen. The average amino acidic profile, obtained averaging the amino acidic profiles of all the samples analyzed, is shown in Table 1, with the confidence interval (α = 0.05).

**Table 1:** Average Amino Acidic Profile of All the Samples Analyzed and Relative Confidence Interval (α = 0.05).

amino acid	relative content (%)	confidence interval
Ala	9.8	±0.4
Gly	27.6	±1.0
Val	2.4	±0.2
Leu	3.1	±0.2
Ile	1.5	±0.2
Met	0.6	±0.1
Ser	4.8	±0.5
Pro	16.2	±0.6
Phe	2.0	±0.1
Asp	7.5	±0.5
Glu	11.2	±0.7
Hyp	12.5	±1.2
Tyr	0.7	±0.4

As expected, Gly is the most abundant amino acid, followed by Pro and Hyp, and in general, very little variability is observed.

The amino acid profile of fish glue (FM) compared with all other glues, which are obtained from mammals, is interesting. Amino acids responsible for the formation of stabilizing H-bridges in the triple helix are more abundant in mammalian collagen than in that of marine species.^6^ The FM sample has the lowest relative amount of polar amino acids (Pro, Hyp, Asp, Glu, Ser), whose total is around 45%, while all other glues have values between 50% and 55%.

#### Proteomics

Proteins were identified in the set of 19 animal glue samples by a shotgun proteomics approach by LC-MS/MS, allowing to molecularly establish the glue source as well as distinguishing between hide and bone glues (details in Tables S3–S21). Identification of collagen alpha-1(III) is indicative that the adhesive was produced from soft connective tissues and skin, as this molecule, together with collagen alpha-1 and −2(I) that are ubiquitous in all the collagen-bearing tissues, is abundant in these tissues, while it is poorly synthetized in bones.^21^ While hide glues are primarily derived from bovine skins and smaller mammals, and sometimes connective tissue, bone glues are predominantly prepared with fresh or extracted bones (degreased and demineralized) from cattle and pigs.^6^

In a preliminary search in the SwissProt database with Chordata as taxonomic restriction, collagen type I was identified in all the samples and collagen type III in some of them, allowing therefore the classification of the samples in hide and bone proteins. It is worth noting that in the BM2 sample, bovine alpha S1 casein was confidently identified (Table S9), indicating the copresence of some milk glue. However, the overall amino acidic profile of this sample, together with the detection of only 3 peptides of alpha S1 casein, while all other milk proteins remained undetected, clearly suggests that the animal glue in BM2 is mainly collagen based.

A straightforward species determination of collagen is however hampered by several factors: the intrinsic simplicity of collagenic protein sequence (which is a hallmark of collagen); the extremely high sequence similarity among the species because of the high degree of evolutionarily conservation; protein sequences of some species of interest to conservation science are either missing in common databases or covered only partly. As a result, although assessing the presence of collagen is relatively easy, in some cases, the discrimination between two animal species (even i.e., between bovine and porcine collagen, for instance) could be quite challenging, since it relies only on the detection of a very few unique peptides.

To simplify species assignment, the search space was reduced to sequences of collagen type I and III of the common domesticates generally used for animal glues, and ultimate searches were carried out using a homemade database, which we named COLLE (60 sequences; 88 859 residues).

Identified collagen chains are summarized in table 2, and the details of protein identifications are provided in Tables S3–S21. Moreover, glues were classified as pure and mixed, when more than a single organism origin was clearly identified. As a result, 13 samples are hide glues (8 pure, HP, and 5 mixed, HM), and 5 are bone glues (all mixed, BM). No pure glue made from bone was identified, and, in our set, fish collagen was confidently identified only in a single sample (FM).

**Table 2:** Collagen Chains Identified in the Animal Glue Samples by LC-MSMS^a^

sample	label on the basis of protein content^b^	taxonomy	collagen α1(I)	collagen α2(I)	collagen α1(III)
rabbit glue SOB1	HP1	*Oryctolagus cuniculus*	yes	yes	yes
rabbit glue SOB2	HP2	*Bos taurus*	yes	yes	yes
rabbit glue SOB3	HP3	*Bos taurus*	yes	yes	yes
rabbit glue SOB4	HP4	*Bos taurus*	yes	yes	yes
rabbit glue SOB5	HM1	*Bos taurus*	yes	yes	yes
		*Sus scrofa*	yes	yes	yes
rabbit glue 10	HP8	*Bos taurus*	yes	yes	yes
rabbit glue 2	HM2	*Oryctolagus cuniculus*	yes	yes	
		*Ovis aries*		yes	yes
		*Capra hircus*	yes		
rabbit glue 3	HM3	*Bos taurus*	yes	yes	yes
		*Oryctolagus cuniculus*	yes	yes	yes
		*Ovis aries*		yes	
rabbit glue 7	HM5	*Oryctolagus cuniculus*	yes		
		*Sus scrofa*	yes	yes	yes
rabbit glue 6	HM4	*Bos taurus*	yes	yes	yes
		*Oryctolagus cuniculus*	yes	yes	
fish glue SOB6	HP5	*Sus scrofa*	yes	yes	yes
fish glue 4	HP6	*Bos taurus*	yes	yes	yes
fish glue 5	HP7	*Bos taurus*	yes	yes	yes
sturgeon fish glue SOB7	FM	*Scyliorhinus canicula*	yes	yes	
strong glue SOB8	BM1	*Sus scrofa*	yes	yes	
		*Bos taurus*	yes	yes	
		*Sus scrofa*	yes	yes	
strong glue SOB9	BM2	*Bos taurus*	yes	yes	
		*Sus scrofa*	yes	yes	
		*Equus asinus*	yes	yes	
strong glue 8	BM4	*Bos taurus*	yes	yes	
		*Sus scrofa*	yes	yes	
		*Equus asinus*		yes	
strong glue 9	BM5	*Bos taurus*	yes	yes	
		*Sus scrofa*	yes		
		*Ovis aries*	yes	yes	
		*Equus asinus*	yes	yes	
strong glue 1	BM3	*Bos taurus*	yes	yes	
		*Sus scrofa*		yes	
		*Equus asinus*	yes	yes	

a Raw data were searched by Mascot MS/MS Ion search using the homemade COLLE database. Details of the identifications are reported in the Supporting Information.

b Protein identification was used to classify glue samples as bone glues (B) and hide glues (H) and further subdivide them into pure (P) and mixed (M).

Although the label “rabbit glue”, would suggest that rabbit skin glues should be produced purely from rabbit skins, we identified the collagen from *Oryctolagus cuniculus* only in five samples (HP1, HM2, HM3, HM4, and HM5), and actually only the sample originally labeled as *Rabbit glue* (SOB1), namely HP1, appears to be a truly pure rabbit glue. As reported by Schellmann,^6^ suppliers might mix rabbit skin glue with bovine hide glue to alter its properties. All the claimed rabbit glues but HP1 contain some bovine and/or sheep collagen, and the sample HM5 contains mainly porcine glue. All the rabbit glues are hide glues since collagen type III was identified.

The samples tagged as bone glues were consistent with their labels since no collagen of type III was detected. Specifically, all of them are mixtures of bovine with porcine collagen, although in some cases, donkey collagen was also identified. Protein identification details of the single samples are reported in the Supplementary Information (Tables S3–S21).

Rather surprisingly, among the samples labeled as fish glues, only in *Sturgeon glue* did we identify some fish collagen, although not from a member of the Acipenseridae family (to which sturgeon species belong) but from the Scyliorhinidae family, as the confident identification of collagen type I *of Scyliorhinus canicula* suggests.

Following the identifications, samples were clustered and relabeled on the basis of the proteins content as Bone glues (B), Hide glues (H), and subdivided in Pure (P) and Mixed (M), as reported in Table 2.

### Collagen Modification: Backbone Cleavage

Animal glue properties, namely, gel strength and viscosity, are influenced by the molecular weight of the constituent protein fragments generated during the material treatment.^6^ A preliminary picture of the molecular weight distribution was obtained by running the acid-soluble collagen fractions on denaturing gel electrophoresis, while some molecular details on the occurrence of polypeptide backbone cleavages was provided by the analysis of the incidence of nonenzymatic hydrolysis sites.

**Figure 1:**
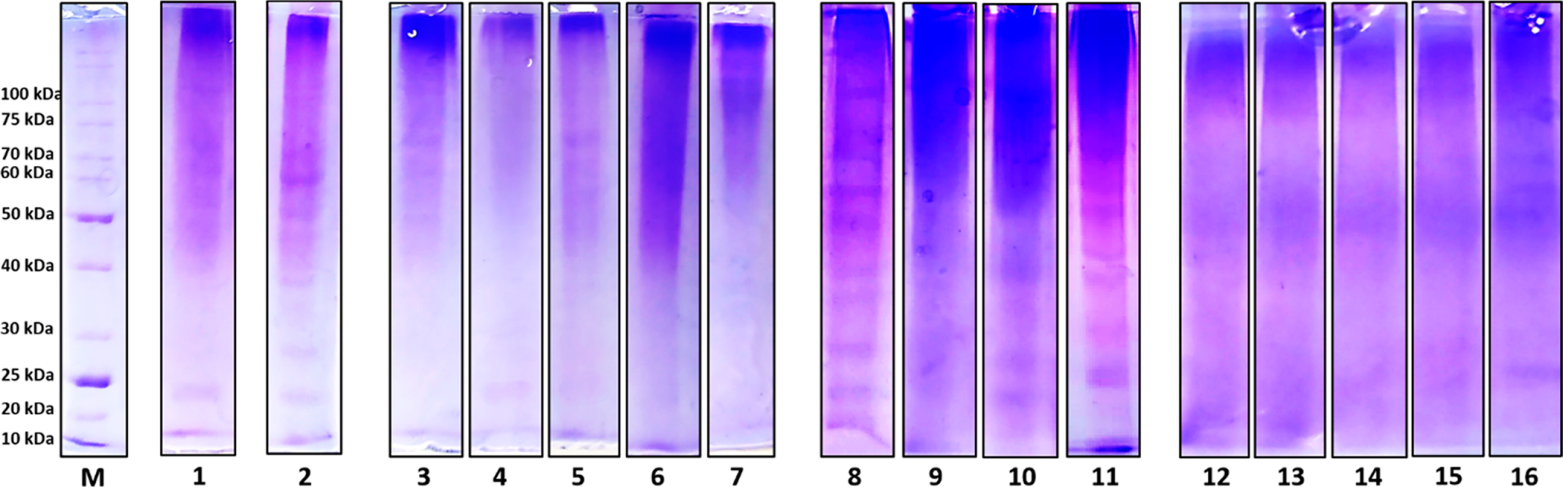
Image of SDS-PAGE of the acid soluble collagen (ASC) fractions prepared from the animal glue samples. Proteins were stained with Coomassie Brilliant Blue (CBB). M: molecular weight markers; Pure rabbit glue: 1: HP1.Pure porcine glue: 2: HP5. Pure bovine glues: 3: HP8; 4: HP2; 5: HP6; 6: HP3; 7: HP4. Mixed animal hide glues: 8: HM1; 9: HM3; 10: HM4; 11: HM5. Mixed animal bone glues: 12: BM3; 13: BM5; 14:BM4; 15: BM1; 16: BM2.

Collagen is a quite challenging material for SDS–PAGE because of its gelling properties, its high molecular weight, and the high occurrence of interchain cross-links impairing protein migration and separation. In the perspective of looking at the occurrence of relatively low-molecular-weight bands (below 100 kDa), we focused on readily soluble species and extracted acid soluble collagen (ASC) fractions following the protocol reported in Hong et al. 2017.^16^Figure 1 shows the ASC different patterns of the samples that were grouped as pure and mixed collagen hide and bone glues, according to the protein identifications reported above. In particular, mixed-collagen hide glues exhibit a higher number of discrete bands with respect to pure hide and mixed-collagen bone glues, or to the pure rabbit glue sample, an artisanal animal glue. This might suggest a less random backbone cleavage (discrete bands rather than a continuous smear), with a residual structural effect behind that deserves further analysis in the future.

**Figure 2:**
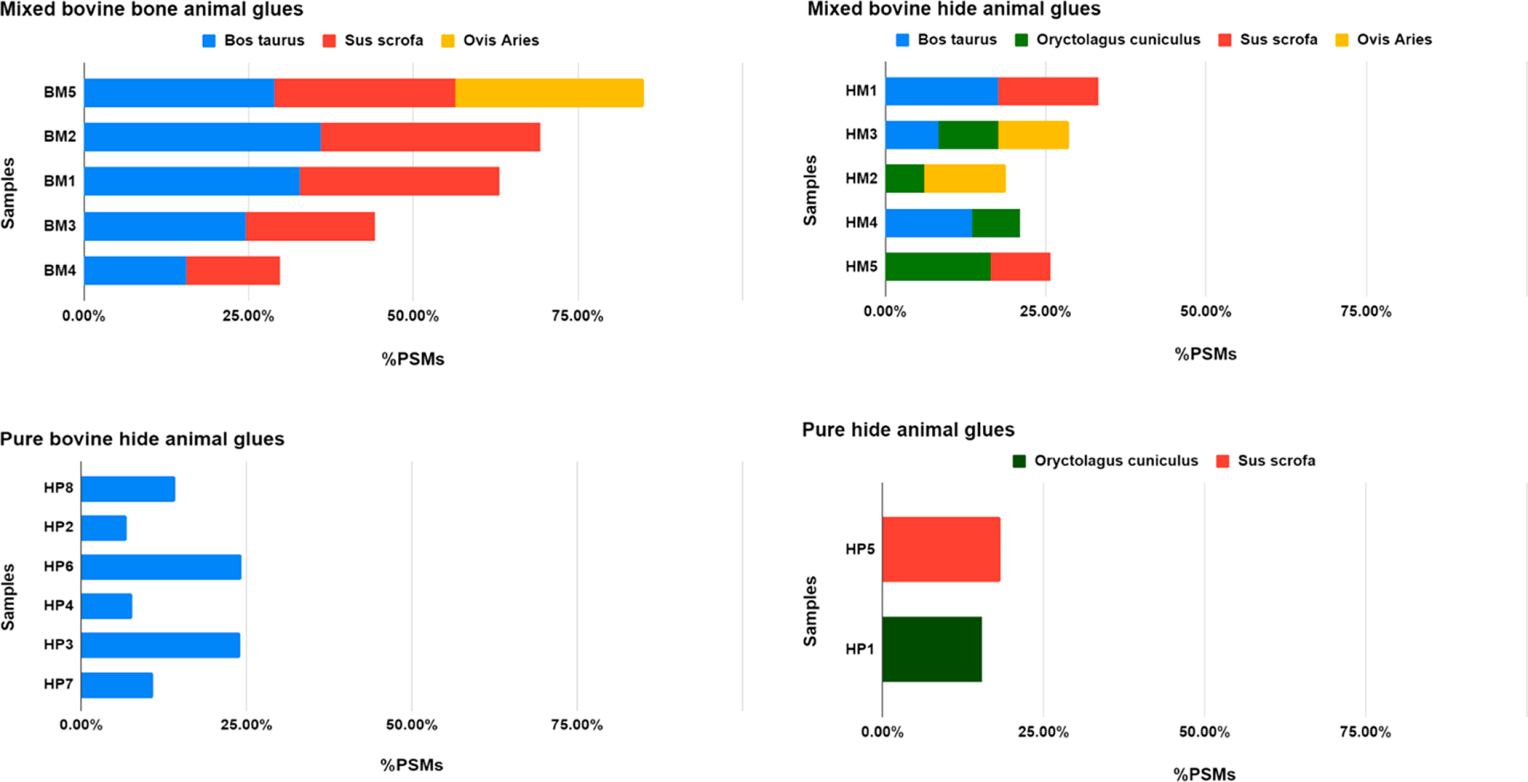
Occurrence of backbone cleavage in animal glue samples. The occurrence of cleavages was semiquantitatively evaluated by calculating the PSMs of semitryptic peptides normalized by the total number of PSMs for the chain (tryptic plus semitryptic). Mixed bovine bone animal glues: BM5, BM2, BM1, BM3, BM4. Mixed bovine hide animal glues: HM1, HM3, HM2, HM4, HM5. Pure bovine hide animal glues: HP8, HP2, HP6, HP4, HP3, HP7. Pure hide animal glues: HP5, HP1.

Backbone cleavage of the polypeptide chain is an expected degradation feature in proteins^22−25^ and is expected in animal glues: collagen is insoluble in cold water and is transformed into soluble gelatin by denaturation and partial hydrolysis, which is achieved by hot water extraction (hydrolytic breakdown).^6^ Such damage at the backbone can be evaluated as semitryptic peptides that will be generated upon trypsin hydrolysis, with a trypsin cleavage site only at one end.^17,26^ The occurrence of semitryptic peptides was semiquantitatively evaluated by counting peptide to spectrum matches (PSMs) and dividing the PSMs of semitryptic peptides with the total PSMs of identified peptides, including both tryptic and semitryptic peptides. We evaluated the backbone cleavage occurrence in the single collagen chains in all the bone and hide samples. As shown in Figure 2, bone glues appear broadly more fragmented in comparison to hide glues. Furthermore, interestingly, more semitryptic peptides were identified in mixed-collagen glues than in pure animal glues.

The data presented so far relate to the portion of the proteins that become soluble after the treatment necessary for the proteomics analyses. To further investigate the degree of backbone cleavage, gaining information on the totality of the samples, analytical (flash) pyrolysis coupled with gas chromatography–mass spectrometry was carried out on all samples.

Pyrolysis products were identified with the help of the NIST20 mass spectral library and by comparison with mass spectra reported in the literature,^27^ and are listed in Table S22. Figure S1 shows the pyrogram of HP6, as an example.

The most characteristic pyrolysis products of proteins are cyclic dipeptides, 2,5-diketopiperazines (DKPs).^20^ Their formation is hypothesized to be a depolymerization involving the cyclization of neighboring amino acids in a polypeptide chain.

In all chromatograms the most intense peak is ascribable to DKP Cyclo (Pro-Gly) (#10, Figure S1). This in fact originates from the cyclization of proline and glycine which are two of the most abundant amino acids present in the collagen chain. Another DKP detected with a high abundance is Cyclo (Pro-Hyp) (#12, Figure S1) and is an identifying marker of collagen. In addition to DKPs, aromatic compounds are also detected, as expected in the pyrolytic profile of animal glue.^20^

All samples were also analyzed by pyrolysis with *in situ* sialylation in order to detect the presence of fatty acids, lipids, and saccharide additives. The data clearly show that fatty acids are present in significant amounts only in samples HP2, HM3, HP1, and HM2. With the exception of rabbit skin animal glue, which has comparatively high fat levels of around 5%,^6^ other glues are at times added with fatty acids to reduce the surface tension and thus to improve wetting and prevent foaming.^6^ HM3, HP1, and HM2 contain animal glue extracted from rabbit, while HP2 is pure bovine glue, indicating that at least in this sample, fatty acids were actually added to the glue and not present as natural components. No other lipid material nor saccharides were detected in the rest of the samples.

To compare the pyrolytic profiles of the different samples, a semiquantitative analyses of DKPs’ was carried out. In particular, the areas of all DKPs detected were normalized for the sample weight (average values of three replicate measurements, RSD < 15%), and the sum of the resulting values are shown in Figure S2.

As DKPs are produced by depolymerization of the polypeptide chain, a relatively high yield of DKPs might be ascribed to a high degree of hydrolysis of the protein. In general, all the bone glue samples present a relatively higher yield of DKPs. This is in agreement with the proteomics data on the soluble fraction and the fact that bone glues have generally a lower molecular weight when compared with hide glues.^1^ Furthermore, the electrophoresis carried out on these samples shows a wider molecular weight distribution for the bone glues, resulting in a smear of bands, thus suggesting a general more randomized degree of hydrolysis. Similarly, the hide glues generally present a lower yield of DKPs, and in fact they exhibit a lower molecular weight distribution in the electrophoresis analysis. Sample HP7, HP8, and FM also present a relatively high yield of DKPs. The position of fish glue in the group with the higher yield of DKPs is not surprising, as glues derived from fish cleave more easily on extensive heating which is needed during the glue preparation procedure.^6^

**Figure 3:**
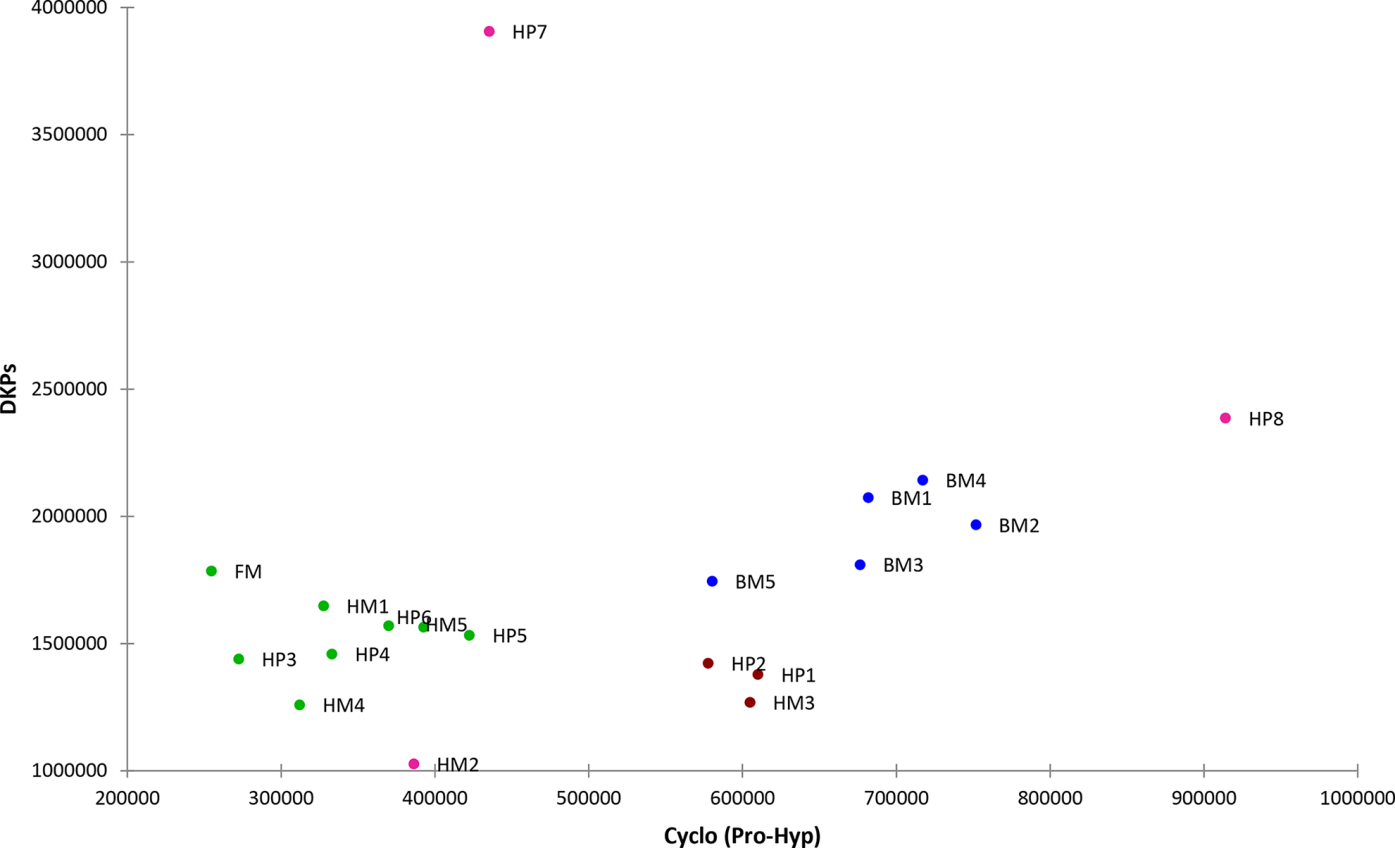
Plot of sum of the areas of all the DPKs versus the area of DPKs Cyclo (Pro-Hyp)

The sum of the areas of all the DPKs was also plotted versus the area of the DPKs Cyclo (Pro-Hyp), and the graph obtained is shown in Figure 3.

The plot shows two main regions. Samples located on the right side of the graph present a relatively higher yield of cyclo (Pro-Hyp) during pyrolysis. All bone glue samples are located on the right of the graph. Samples HP2, HM3, and HP1 are tightly grouped together on the right side of the graph. Their position could be influenced though by the presence of fatty acids in the sample, which might affect the mechanism of formation of diketopiperazines. Samples HP7 and HP8 are quite separated from the rest of the graph, both presenting a high reaction yield of DKPs, and sample HP8 shows also a high yield of Cyclo (Pro-Hyp). The relatively high yield of cyclo (Pro-Hyp) could be due to a relatively high concentration of hydroxyproline in the polypeptide chain. Although, generally, samples presenting a higher yield of cyclo (Pro-Hyp) also present a relatively higher content of Hyp, as determined by the GC-MS analyses, this is not strictly true for all the samples (BM2 for example has a low Hyp content, while sample HP3 has a high Hyp content). Another factor that could affect the yield of cyclo (Pro-Hyp) could be the structural arrangement of the polypeptide chain: hydrogen bonds might favor other pyrolytic processes with respect to depolymerisation, as already shown for ovalbumin.^28^ If this is the case, data would suggest that in the bone glue samples hydroxyproline is less involved in hydrogen bonds and, thus, that polypeptide chains are less tightly organized with respect to the hide glue samples. More research is necessary to clarify this point.

### Collagen Modification: Deamidation of Glutamine and Asparagine

Charge distribution in collagen chains is reported to affect animal glue properties, like dependence of viscosity on pH.^6^ During glue processing, the acidic or alkaline treatments favor hydrolysis of the amide groups in collagen to a greater or lesser extent, by deamidating the lateral chain of glutamine and asparagine, thus affecting the isoelectric point by unlocking charged functions^2,6^ and by hydrolyzing the peptide bond. Bones and tissues are both subject to alkaline and acidic treatments, to remove unwanted material and to initiate the protein denaturation, making the protein available for extraction in hot water in the following stages.^29,30^ Deamidation of glutamine and asparagine residues is a nonenzymatic modification that can be followed by mass spectrometry. It results in a +0.98402 mass shift as a consequence of the conversion of the polar, noncharged side-chain amide group to the carboxylate moiety.

We evaluated deamidation occurrence in the set of samples keeping in mind that deamidation is routinely searched for in aged proteins^31,32^ and viewed as a global indicator of sample preservational quality,^33^ since rates and levels of deamidation are affected by several chemical and environmental factors. Harsher conditions of collagen extraction in the preparation of glues might have been imprinted also in the profile and level of collagen deamidation.

Deamidation was evaluated from raw LC-MS/MS data by MaxQuant software with an in-house script based on peptide spectrum matches intensities for semiquantitative evaluation, as reported in.^31^ Asparagine (Asn) deamidation is faster than glutamine (Gln) deamidation and therefore usually less informative,^33^ and consequently, although we always report deamidation at both, our considerations mainly refer to glutamine deamidation.

**Figure 4:**
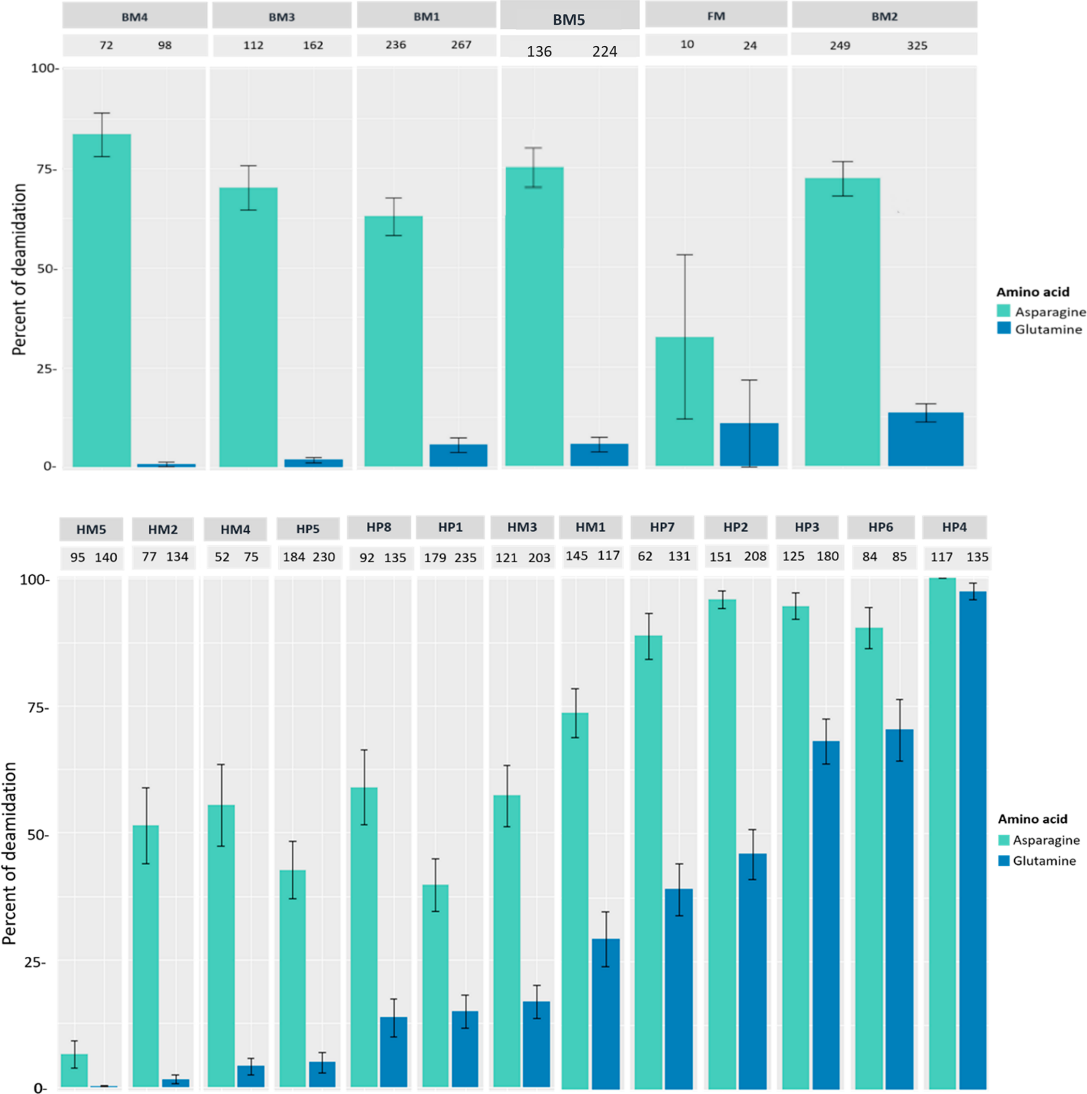
Overall percentage of deamidation for asparagines (N) and glutamines (Q) residues for the collagen chains identified in the bone (upper panel) and hide (lower panel) glue samples. Error bars represent standard deviation and numbers above each bar represent the number of peptides the data is based on.

The deamidation levels of the glue samples are reported in figure 4. Deamidation levels are extremely variable, with deamidation of hide glues being extremely variable, ranging between 2 and 98%. It is possible to observe that, on average, bone glues are less deamidated, with values ranging between 2 and 12%.

It is interesting to observe that, among the hide glues, the most deamidated are those that contain only bovine collagen (HP2, HP3, HP4, HP6). To further investigate this feature, we evaluated the deamidation level in the single collagen chains of glues that contain collagen from a single animal species. Figures S3 and S4 show the deamidation levels in the collagen chains in the pure bovine glues, pure rabbit, and pure porcine glues, respectively. All the collagen chains of the pure bovine glues are extensively deamidated in comparison to the porcine (Figure S4A) and rabbit (Figure S4B) ones. The deamidation level of the bovine collagen chains in the mixed glues is then reported in Figure S5. The comparison of the values in Figures S3 and S5 suggests that bovine chains are most prone to deamidation, or that the procedures used to extract the glue from bovine hides promote a more extensive deamidation. It has been reported that acid-processed glue brings to little amide group modification, while alkaline-processed glue is characterized by extensive hydrolysis of the amide groups. Harsh alkaline treatments at relatively high temperatures^2^ are carried out on chromium tanned leather wastes, to remove chromium and separate the collagen to be used for the glue production.^34^ A possible explanation of the higher degree of deamidation observed in pure bovine glues could be the fact that bovine skins used in their production derive from leather wastes. More research is necessary to understand this point.

As a further investigation, we manually combined the peptides on the basis of the protein family regardless of the animal species in mixed animal glue samples. Figures S6–S8 report the deamidation of the three more abundant protein families: collagen-type I chain 1, collagen-type I chain 2, and collagen-type III chain 1. From the comparison of the S3A and S6 we can observe that collagen-type I chain 1 in the mixed glues are less deamidated than in the pure bovine glues. This observation is confirmed also from the comparison of the other protein families of mixed glues with the same protein families of the pure glues: Figure S7 versus Figure S3B and Figure S8 versus Figure S3C. Mixed-collagen glues are overall less deamidated than pure glues (see also Figure 4).

Furthermore, we examined site-specific deamidation distribution (i.e., whether deamidation preferentially occurs in specific positions in the protein chain) in the animal glue samples. Attention was primarily focused to the pure bovine animal glues only, to avoid the ambiguity of homologous peptides of collagen from different species. A site-specific evaluation of deamidation (NQ) patterns was performed manually by checking the fragmentation spectra of the peptides containing Asn and Gln.

We classified the single positions of asparagines and glutamines along the collagen sequences as those detected only as unmodified (X), those detected only as deamidated (D), those that were identified both as deamidated and unmodified (DX) and, finally, those that were not detected at all, nor unmodified nor deamidated (NF) (see Tables S23 and S24). This preliminary first and simple approach provides a glance on deamidation occurrence in the main chains of collagen α1(I) and collagen α2(I).

As shown in Tables S23 and S24, almost all of the glutamines and asparagines positions that have been identified underwent some deamidation, that means that were detected either only as deamidated or both deamidated and unmodified. It is worth mentioning that some glutamines and asparagines are in regions that have not been covered in the identification. Interestingly, our experiments, at least at this level, where only detection of deamidated/unmodified was considered, do not point out any marked difference among the samples, and deamidation seems to be spread along the sequences, without any hot spot, suggesting the absence of any three-dimensional effect. This might not come as a surprise, if we consider that, in the glues, collagen is denatured, as a consequence of the extraction procedure, and, actually, the acidic and alkaline treatments are specifically used to break intermolecular and intramolecular bonds, thus exposing the whole polypeptide chain to the aqueous environment. This could eventually make a marked difference in respect to the situation observed with ancient collagen from bone, where three-dimensional structural effects seem to play a significant role on deamidation.^35^

Furthermore, it is interesting to note that almost all the methionine detected were found as oxidized (see Tables S23 and S24). For instance, in the collagen I chain a1 the methionine in positions 300, 579, 728 and M were detected always and only oxidized. Similarly, in collagen type I chain 2 the M in position 445 was detected always as oxidized, with all the other positions belonging to regions that were not covered at all in the identification. Site-specific methionine oxidation trend is the same regardless the sample preparation of the single animal glue.

## Conclusions

This work focused on the molecular characterization of a series of collagen-based animal glues produced for restoration purposes. Molecular weight distribution, stabilization of the protein matrix by hydrogen bonding, charge distribution, all have an impact on the performances of the glue and are determined by amino acid composition, but also and most importantly by chemical modifications occurring during preparation procedures. Although much attention has been devoted to physical properties of collagen based animal glues,^2,6,36,37^ a systematic characterization of molecular properties of glues is still lacking.

The most striking feature we found was the fairly common discrepancy in commercial glues between label and actual animal origin. A few samples were made of tissues from a single organism, and a classification in pure and mixed, hide and bone glues was made on the basis of proteomic results. Collagen type III was actually identified in all the hide glues, confirming the use of skin and cartilaginous tissue, and thus its identification can be safely proposed as an effective analytical molecular marker to discriminate between hide and bone glues. This important quality parameter can be unequivocally assessed by the described proteomic analysis.

MS/MS data were used to investigate collagen degradation in animal glue. Modifications of amino acids, such as oxidation of methionine, deamidation of asparagine and glutamine, as well as backbone cleavage, were evaluated since they affect charge and molecular weight distributions.^6^ This is the first detailed analysis on the occurrence of deamidations in a large and diversified set of collagen-based animal glues, and this modification is expected to strongly influence the rheological properties of the adhesive material since it changes collagen pI. Bone glues are on average less deamidated than hide glues. Exhaustive evaluation of deamidation levels is made difficult by the presence of multiple collagens in mixture in the mixed glues. We therefore collectively calculated deamidation for each family of collagen proteins in single samples (i.e., all type I, chain alpha 1 collagen sequences) and, interestingly, mixed-collagen animal glues were collectively found less deamidated than pure animal glues. Furthermore, the collagen chains of *Bos taurus* in hide-mixed glues are significantly less deamidated than the same chains in pure bovine glues. A possible explanation can be found in the origin of the hides used for the glue preparation, which might derive from tannery wastes.

Backbone cleavage, causing a partial depolymerization of collagen chains, is also a degradation feature that is expected to occur upon extreme pH treatments and extended heating during glue preparation. It is a fact that the resulting molecular weight distribution is considered among the most critical parameters determining glue properties.^6^ Assessing the molecular weight distribution of fragmented collagen is not an easy task due to the intrinsic fibrous properties of collagen, and further complicated by the extensive network of intermolecular cross-links that are essential in providing the connective tissues with their stability, cohesiveness, and physicochemical properties.^38^ The number of semitryptic peptides is connected to backbone cleavages and was higher in the bone glues than in the hide ones. This result agrees with the pyrolysis data, showing a higher yield of DKPs upon thermal degradation in the bone glues, and with the known generally harsher conditions used to extract collagen from bones.

Data herein presented confirm the heterogeneity of collagen-based animal glues at the molecular level, heterogeneity strongly increased by the preparation and manufacturing procedures, affecting the properties of the glue, possibly more than the collagen origin itself. These data, showing that, on average, bone glues are less deamidated than hide glues, but more fragmented, and mixed-collagen glues are overall less deamidated than pure glues, pave the way to a correlation between molecular modifications and material performances in animal glues. Moreover, this original analytical characterization will be useful in a wider, comparative perspective aimed at the characterization of the variety of collagen-based materials.

## Supporting Information Available

Animal glues’ information, provenience, and classification upon proteomic analysis results (Table S1); relative content of amino acids obtained by GC-MS (Table S2); proteomic identification details (Tables S3–S21); backbone cleavage by (flash) pyrolysis GC-MS (Figures S1–S2, Table S22); deamidation of collagen (Figures S3–S8, Tables S23–S24); accession entries in COLLe database (Table S25) (PDF)
