# Supplementary material for: Proteomic Characterization of Collagen-Based Animal Glues for Restoration
Source: J Proteome Res. 2022 Aug 15;21(9):2173–84. doi: 10.1021/acs.jproteome.2c00232 (PMC9442796; doi:10.1021/acs.jproteome.2c00232)
Supplement: Supplementary file 1 — pr2c00232_si_001.pdf [file pr2c00232_si_001.pdf]

Supplementary materials

Proteomic characterization of collagen-based animal glues for restoration.

Georgia Ntasi[1], Sara Sbriglia[1], Rossana Pitocchi[1], Roberto Vinciguerra[1], Chiara Melchiorre[1], Laura Dello Ioio[2], Giancarlo Fatigati[3], Emanuele Crisci[4], Ilaria Bonaduce[4], Andrea Carpentieri[1,3,5], Gennaro Marino[1,3] and Leila Birolo[1,5]*.

[1] Department of Chemical Sciences, University of Naples Federico II, Complesso Universitario di Monte S. Angelo, Via Cinthia 21, 80126 Naples, Italy
[2] Dello Ioio Restauri, 80069 Vico Equense, Naples, Italy
[3] Department of Humanities, University Suor Orsola Benincasa, via Santa Caterina da Siena 37, 80132, Naples, Italy
[4] Department of Chemistry and Industrial Chemistry, via Risorgimento 35, University of Pisa, 56126 Pisa, Italy
[5] Task Force "Metodologie Analitiche per la Salvaguardia dei Beni Culturali", University of Naples Federico II, 80138 Naples, Italy

*Correspondence to:

Leila Birolo, Department of Chemical Sciences, University of Naples Federico II, Complesso Universitario di Monte S. Angelo, Via Cinthia 21, 80126 Naples, Italy. Phone: +39-081-679939. E-mail: birolo@unina.it.

Table of contents

Table of contents 1
Materials and methods 3
Animal glue samples. 3
Table S1: Animal glue's information, provenience, and classification upon proteomic analysis results.. 3
Results 6
Table S2 Relative content of amino acids obtained by GC-MS. 6
Proteomic identification 7
Table S3: Collagen chains identified in the Rabbit Glue SOB1 sample (HP1) 7
Table S4: Collagen chains identified in the Rabbit Glue SOB5 sample. (HM1) 7
Table S5: Collagen chains identified in the Rabbit Glue SOB2 sample. (HP2) 7
Table S6: Collagen chains identified in the Rabbit Glue SOB3 sample. (HP3) 7
Table S7: Collagen chains identified in the Rabbit Glue SOB4 sample (HP4). 7
Table S8: Collagen chains identified in the Strong glue SOB8 sample (BM1). 8
Table S9: Proteins identified in the Strong glue SOB9 sample (BM2) 8

Table S10: Collagen chains identified in the Fish Glue SOB6 sample (HP5) 8
Table S11: Collagen chains identified in the Sturgeon fish glue SOB7 sample. (FM) 8
Table S12: Collagen chains identified in the Strong Glue 1 sample. (BM3) 9
Table S13: Collagen chains identified in the Rabbit Glue 2 sample. (HM2) 9
Table S14: Collagen chains identified in the Rabbit glue 3 sample (HM3). 9
Table S15: Collagen chains identified in the Fish glue 4 sample (HP6). 9
Table S16: Collagen chains identified in the Fish glue 5 sample (HP7). 10
Table S17: Collagen chains identified in the Rabbit glue 6 sample (HM4). 10
Table S18: Collagen chains identified in the Rabbit glue 7 sample (HM5). 10
Table S19: Collagen chains identified in the Strong Glue 8 sample (BM4). 10
Table S20: Collagen chains identified in the Strong Glue 9 sample (BM5). 11
Table S21: Collagen chains identified in the Rabbit Glue 10 sample (HP8). 11
Collagen modification: Backbone cleavage 12
Figure S1. HP6 glue pyrogram. Peak numbering refers to compounds listed in Table S22 12
Table S22 Molecules identified in the pyrogram of HP6 and their most abundant mass spectrometric ions (*m/z*). 12
Figure S2. Sum of the areas of all DKPs detected normalized for the sample weight 13
Collagen damage: deamidation of glutamine and asparagine. 14
Figure S3: Overall percentage of deamidation of the single collagen chains in bovine pure hide glues (HP). 14
Figure S4: Overall percentage of deamidation of the single collagen chains in pure hide glues that contain only porcine glue (HP5) (A panel) and only rabbit glue (HP1) (B panel). 15
Figure S5: Overall percentage of deamidation of bovine collagen alpha-1(I) (A), collagen alpha-2(I) (B), and collagen alpha-1(III) (C) chains in mixed hide and bone glues containing bovine collagen. 15
Figure S6: Overall percentage of deamidation of the protein family collagen alpha-1(I) in mixed animal glues. 16
Figure S7: Overall percentage of deamidation of the protein family collagen alpha-2(I) in mixed animal glues. 16
Figure S8: Overall percentage of deamidation of the protein family collagen alpha-1(III) in mixed animal glues. 17
Table S23: Site-specific evaluation of deamidation (N, Q) and oxidation (M) along *Bos taurus* collagen α1(I) sequence in all the pure bovine animal glues. 18
Table S24: Site-specific evaluation of deamidation (N, Q) and oxidation (M) along *Bos taurus* collagen α2(I) sequence in all the pure bovine animal glues. 19
Table S25. Accession entries as in the COLLE database and their correspondence in Uniprot database .. 21
References 22

Materials and methods

Animal glue samples.

Table S1: Animal glue's information, provenience, and classification upon proteomic analysis results

Animal glue Name	Age	Source	**H**ide **P**ure (**HP**)<br>**H**ide **M**ixed (**HM**)<br>**B**one **M**ixed (**BM**)<br>**F**ish **M**ixed (**FM**)
Rabbit glue SOB1 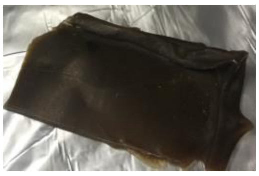	After first World war	Suor Orsola Benincasa	**HP1**
Rabbit Glue SOB2 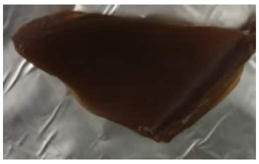	1960s	Suor Orsola Benincasa	**HP2**
Rabbit glue SOB3 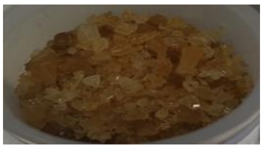	1960s	Suor Orsola Benincasa	**HP3**
Rabbit glue SOB4 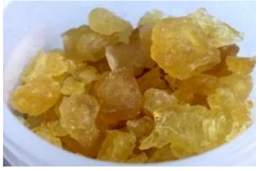	1960s	Suor Orsola Benincasa	**HP4**
Rabbit glue SOB5 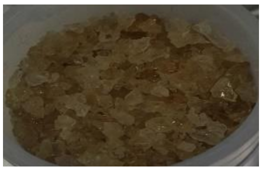	1960s	Suor Orsola Benincasa	**HM1**
Rabbit glue 10 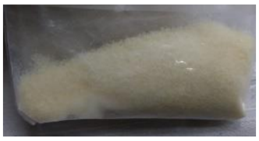	Modern	Museo Nacional del Prado	**HP8**

Rabbit glue 3 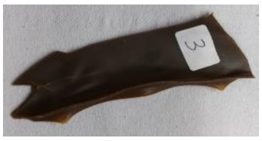	Modern	Museo Nacional del Prado	**HM3**
Rabbit glue 7 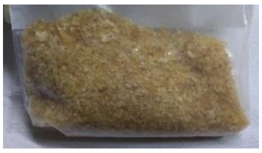	Modern	Museo Nacional del Prado	**HM5**
Rabbit glue 6 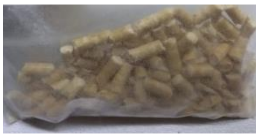	Modern	Museo Nacional del Prado	**HM4**
Fish glue SOB6 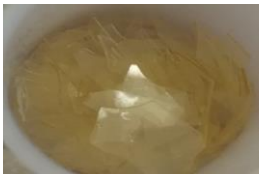	1960s	Suor Orsola Benincasa	**HP5**
Fish glue 4 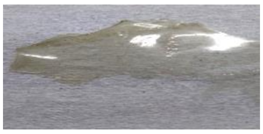	Modern	Museo Nacional del Prado	**HP6**
Fish glue 5 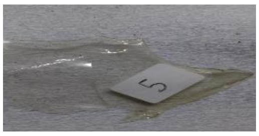	Modern	Museo Nacional del Prado	**HP7**
Sturgeon fish glue SOB7 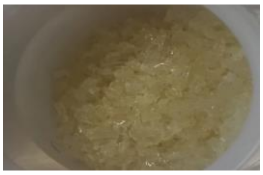	1960s	Suor Orsola Benincasa	**FM**
Strong glue SOB8 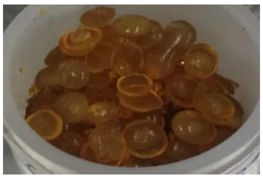	1960s	Suor Orsola Benincasa	**BM1**

Strong glue SOB9 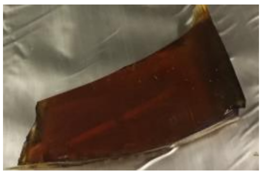	1960s	Suor Orsola Benincasa	**BM2**
Strong glue 8 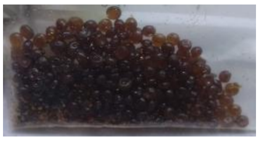	Modern	Museo Nacional del Prado	**BM4**
Strong glue 9 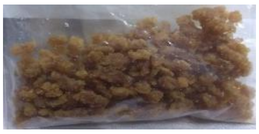	Modern	Museo Nacional del Prado	**BM5**
Strong glue 1 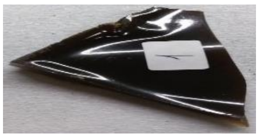	Modern	Museo Nacional del Prado	**BM3**
Rabbit glue 2 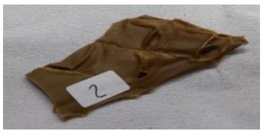	Modern	Museo Nacional del Prado	**HM2**

Results

Table S2 Relative content of amino acids obtained by GC-MS

Sample		Ala	Gly	Val	Leu	Ile	Met	Ser	Pro	Phe	Asp	Glu	Hyp	Tyr
HM2	**average**	**9.9**	**24.9**	**2.8**	**3.8**	**1.7**	**0.8**	**4.8**	**14.8**	**2.6**	**10.4**	**12.6**	**7.1**	**3.7**
	**SD**	0.4	1.0	0.2	0.2	0.1	0.2	0.7	0.1	0.2	0.6	4.2	1.4	3.4
HP4	**average**	**10.9**	**29.2**	**2.6**	**3.4**	**1.6**	**0.7**	**3.9**	**18.4**	**1.7**	**7.3**	**8.7**	**11.1**	**0.4**
	**SD**	0.2	1.9	0.2	0.3	0.1	0.2	0.3	0.8	0.1	0.9	0.3	0.4	0.1
BM2	**average**	**10.4**	**28.7**	**2.7**	**3.4**	**1.5**	**0.5**	**4.8**	**14.7**	**2.4**	**8.1**	**12.1**	**10.0**	**0.6**
	**SD**	1.4	0.1	0.3	0.4	0.2	0.1	1.5	0.2	0.3	0.7	0.9	3.8	0.3
BM1	**average**	**9.6**	**26.8**	**2.5**	**3.0**	**1.3**	**0.4**	**3.8**	**16.2**	**2.2**	**7.7**	**12.4**	**13.3**	**0.7**
	**SD**	0.3	4.9	0.0	0.1	0.0	0.1	0.9	2.4	0.6	3.1	4.5	1.9	0.4
HP8	**average**	**9.3**	**25.2**	**2.5**	**3.0**	**1.4**	**0.5**	**4.9**	**15.2**	**1.8**	**7.3**	**10.5**	**17.8**	**0.5**
	**SD**	0.5	0.0	0.2	0.1	0.1	0.1	0.0	0.8	0.0	0.6	0.9	0.6	0.3
HP1	**average**	**9.1**	**26.8**	**2.5**	**3.0**	**1.4**	**0.4**	**4.5**	**15.9**	**1.8**	**7.1**	**10.9**	**16.3**	**0.5**
	**SD**	2.0	6.7	0.6	0.7	0.3	0.0	1.1	2.4	0.1	0.3	0.3	11.5	0.2
BM5	**average**	**10.1**	**25.5**	**2.8**	**3.6**	**1.6**	**0.6**	**4.6**	**13.7**	**2.5**	**8.1**	**12.8**	**12.8**	**1.4**
	**SD**	0.2	0.5	0.3	0.2	0.1	0.2	0.1	2.3	0.0	0.3	1.4	1.3	0.0
BM3	**average**	**9.8**	**25.0**	**2.7**	**3.5**	**1.5**	**0.6**	**4.0**	**15.2**	**2.3**	**7.8**	**11.7**	**15.0**	**0.8**
	**SD**	0.6	1.5	0.3	0.3	0.1	0.1	0.2	0.3	0.0	0.2	0.6	2.5	0.1
HM3	**average**	**9.6**	**24.8**	**2.7**	**3.3**	**1.6**	**0.5**	**5.4**	**15.4**	**2.2**	**8.8**	**12.2**	**12.7**	**0.7**
	**SD**	0.6	2.1	0.2	0.1	0.1	0.0	0.8	0.8	0.1	0.4	0.6	2.4	0.1
HM1	**average**	**10.4**	**29.4**	**2.4**	**2.9**	**1.5**	**0.3**	**3.8**	**17.3**	**1.9**	**7.3**	**11.5**	**11.0**	**0.3**
	**SD**	0.6	0.7	0.1	0.0	0.0	0.1	0.9	3.0	0.5	2.7	4.4	4.1	0.1
BM4	**average**	**10.8**	**24.8**	**2.9**	**3.4**	**1.6**	**0.6**	**5.6**	**15.8**	**2.0**	**6.4**	**9.4**	**15.8**	**0.9**
	**SD**	1.4	2.9	0.6	0.8	0.3	0.2	2.5	1.9	0.1	0.5	0.1	6.5	0.6
FM	**average**	**11.1**	**31.6**	**2.8**	**3.3**	**2.7**	**1.2**	**4.1**	**15.8**	**1.9**	**5.9**	**9.5**	**9.8**	**11.1**
	**SD**	0.1	3.0	0.1	0.1	0.1	0.5	0.3	0.2	0.0	0.3	0.3	3.1	0.1
HP3	**average**	**9.0**	**26.3**	**2.3**	**3.0**	**1.4**	**0.2**	**4.7**	**17.2**	**1.8**	**7.5**	**11.9**	**14.5**	**0.3**
	**SD**	0.3	0.1	0.3	0.5	0.2	0.2	0.5	1.7	0.1	0.2	1.4	0.5	0.1
HP7	**average**	**9.7**	**27.8**	**2.3**	**2.9**	**1.5**	**0.5**	**4.2**	**15.7**	**2.1**	**7.9**	**12.6**	**12.3**	**0.5**
	**SD**	0.1	2.6	0.0	0.1	0.0	0.1	0.4	2.5	0.4	2.2	3.4	1.8	0.5
HP2	**average**	**9.8**	**28.8**	**1.8**	**3.0**	**1.0**	**0.9**	**4.8**	**17.6**	**1.8**	**6.6**	**9.2**	**14.2**	**0.4**
	**SD**	0.5	4.9	0.1	0.1	0.0	0.0	0.9	2.3	0.2	2.3	2.5	1.8	0.1
HM4	**average**	**10.1**	**29.5**	**1.7**	**3.1**	**1.2**	**0.7**	**4.4**	**16.6**	**2.0**	**7.8**	**11.0**	**11.4**	**0.5**
	**SD**	0.7	6.8	0.2	0.5	0.1	0.0	0.6	2.1	0.3	2.1	3.2	3.8	0.3
HP6	**average**	**9.3**	**28.6**	**1.4**	**2.6**	**1.1**	**0.7**	**6.4**	**15.8**	**2.0**	**7.8**	**12.8**	**11.1**	**0.4**
	**SD**	0.9	0.4	0.2	0.4	0.2	0.0	0.3	1.7	0.1	0.4	0.2	0.9	0.1
HP5	**average**	**9.4**	**28.7**	**1.5**	**2.7**	**0.8**	**0.9**	**5.0**	**14.7**	**2.4**	**7.9**	**13.6**	**11.4**	**0.9**
	**SD**	0.1	2.3	0.0	0.0	0.0	0.0	0.0	2.7	0.2	0.5	0.3	1.3	0.0
HM5	**average**	**7.9**	**29.3**	**2.3**	**2.8**	**1.3**	**0.6**	**8.6**	**18.0**	**1.7**	**6.8**	**10.4**	**10.1**	**0.2**
	**SD**	1.5	5.3	0.3	0.1	0.0	0.2	2.6	0.8	0.5	1.2	3.4	0.0	0.1

Proteomic identification

MS/MS raw data were routinely searched by Mascot MS/MS Ion search software using COLLE database and considering deamidation on Gln and Asn, oxidation on Met, hydroxylation of proline and lysine as variable modifications, unless otherwise stated. LC-MS/MS data and COLLE database have been deposited to Mendeley Data https://data.mendeley.com/datasets/hbmc8yhf7y/4

Table S3: Collagen chains identified in the Rabbit Glue SOB1 sample (HP1)

Gene Name	Taxonomy	Protein Identifier	Protein Score	Sequence Coverage (%)	No. of Peptides	No. of Unique Peptides
COL1A1	*Oryctolagus cuniculus*	G1T4A5	2887	57	47	6
COL1A2	*Oryctolagus cuniculus*	G1T2Z5	2847	55	47	17
COL3A1	*Oryctolagus cuniculus*	G1T8J0	1689	33	32	15

Table S4: Collagen chains identified in the Rabbit Glue SOB5 sample. (HM1)

Gene name	Taxonomy	Protein identifier	Protein Score	Sequence coverage (%)	No. of Peptides	No. of Unique Peptides
COL1A1	*Bos taurus*	P02453	2698	48	48	2
COL1A1	*Sus scrofa*	A0A287A1S6	2492	48	12	5
COL1A2	*Sus scrofa*	A0A1S7J1Y9	2140	45	36	12
COL1A2	*Bos taurus*	P02465	1802	39	18	7
COL3A1	*Bos taurus*	P04258	1119	28	22	7
COL3A1	*Sus scrofa*	F1RYI8	865	23	6	4

Table S5: Collagen chains identified in the Rabbit Glue SOB2 sample. (HP2)

Gene name	Taxonomy	Protein identifier	Protein Score	Sequence coverage (%)	No. of peptides	No. of Unique Peptides
COL1A1	*Bos taurus*	P02453	2704	53	47	1
COL1A2	*Bos taurus*	P02465	2568	54	42	7
COL3A1	*Bos taurus*	P04258	1669	34	27	10

Table S6: Collagen chains identified in the Rabbit Glue SOB3 sample. (HP3)

Gene Name	Taxonomy	Protein Identifier	Protein Score	Sequence Coverage (%)	No. of Peptides	No. of Unique Peptides
COL1A1	*Bos taurus*	P02453	2345	44	43	1
COL1A2	*Bos taurus*	P02465	1781	36	29	6
COL3A1	*Bos taurus*	P04258	622	16	13	10

Table S7: Collagen chains identified in the Rabbit Glue SOB4 sample (HP4).

Gene name	Taxonomy	Protein identifier	Protein Score	Sequence coverage (%)	No. of Peptides	No. of Unique Peptides

COL1A1	*Bos taurus*	P02453	2497	48	47	2
COL1A2	*Bos taurus*	P02465	1647	35	30	4
COL3A1	*Bos taurus*	P04258	925	28	22	6

Table S8: Collagen chains identified in the Strong glue SOB8 sample (BM1).

Gene name	Taxonomy	Protein identifier	Protein Score	Sequence coverage (%)	No. o f Peptides	No. of Unique Peptides
COL1A2	*Bos taurus*	P02465	2555	51	44	9
COL1A1	*Bos taurus*	P02453	2507	44	41	1
COL1A1	*Sus scrofa*	A0A287A1S6	2367	42	16	5
COL1A2	*Sus scrofa*	A0A1S7J1Y9	2292	43	11	9

Table S9: Proteins identified in the Strong glue SOB9 sample (BM2)

MS/MS raw data were searched by Mascot MS/MS Ion search software using COLLE database and considering deamidation on Gln and Asn, oxidation on Met, hydroxylation of proline and lysine as variable modifications. [a]Bovine casein, highlighted in grey, was identified in a preliminary search against SwissProt database (561911 sequences; 202173710 residues) using Chordata as taxonomy (85899 sequences) and considering methionine oxidation and phosphorylation of serine and threonine as variable modifications.

Gene name	Taxonomy	Protein identifier	ProteinScore	Sequence coverage (%)	No. of Peptides	No. of Unique Peptides
COL1A2	*Bos taurus*	P02465	2660	55	45	7
COL1A1	*Bos taurus*	P02453	932	45	42	5
COL1A1	*Sus scorfa*	A0A287A1S6	2250	42	7	4
COL1A2	*Sus scorfa*	A0A1S7J1Y9	2064	40	14	8
COL1A1	*Equus asinus*	B9VR88	2372	43	4	3
COL1A2	*Equus asinus*	B9VR89	2027	45	12	8
CASA1[a]	*Bos taurus*	P02662	552	19	3	3

Table S10: Collagen chains identified in the Fish Glue SOB6 sample (HP5)

Gene Name	Taxonomy	Protein identifier	Protein Score	Sequence coverage (%)	No. of Peptides	No. of Unique Peptides
COL1A1	*Sus scrofa*	A0A287A1S6	3107	54	52	8
COL1A2	*Sus scrofa*	A0A1S7J1Y9	3026	55	47	19
COL3A1	*Sus scrofa*	F1RYI8	1345	33	29	15

Table S11: Collagen chains identified in the Sturgeon fish glue SOB7 sample. (FM)

Gene Name	Taxonomy	Protein Identifier	Protein Score	Sequence Coverage (%)	No. of Peptides	No. of Unique Peptides
COL1A1	*Scyliorhinus canicula*	D0PQF7	586	13	11	8
COL1A1	*Sus scrofa*	A0A287A1S6	425	11	6	3
COL1A2	*Sus scrofa*	A0A1S7J1Y9	205	8	5	4

Table S12: Collagen chains identified in the Strong Glue 1 sample. (BM3)

Gene Name	Taxonomy	Protein Identifier	Protein Score	Sequence Coverage (%)	No. of Peptides	No. of Unique Peptides
COL1A2	*Bos taurus*	P02465	2841	60	58	8
COL1A1	*Bos taurus*	P02453	2735	60	61	1
COL1A2	*Sus scrofa*	A0A1S7J1Y9	1917	47	44	5
COL1A2	*Equus asinus*	B9VR89	1656	43	38	7
COL1A1	*Equus asinus*	B9VR88	2263	51	52	1

Table S13: Collagen chains identified in the Rabbit Glue 2 sample. (HM2)

Gene Name	Taxonomy	Protein Identifier	Protein Score	Sequence Coverage (%)	No. of Peptides	No. of Unique Peptides
COL1A2	*Ovis aries*	M4JBX7	2310	59	52	5
COL1A1	*Capra hircus or Ovis aries*	A0A452FHU9/ W5P481	2308	53	53	0
COL1A1	*Oryctolagus cuniculus*	G1T4A5	2053	60	49	4
COL3A1	*Ovis aries*	W5Q4S0	1254	31	32	7
COL1A2	*Oryctolagus cuniculus*	G1T2Z5	1240	41	31	10
COL3A1	*Oryctolagus cuniculus*	G1T8J0	866	23	22	7

Table S14: Collagen chains identified in the Rabbit glue 3 sample (HM3).

Gene Name	Taxonomy	Protein Identifier	Protein Score	Sequence Coverage (%)	No. of Peptides	No. of Unique Peptides
COL1A2	*Oryctolagus cuniculus*	G1T2Z5	2508	59	51	17
COL1A1	*Capra hircus or Ovis aries*	A0A452FHU9/ W5P481	2386	56	54	2
COL1A1	*Bos taurus*	P02453	2297	54	48	0
COL1A1	*Oryctolagus cuniculus*	G1T4A5	2231	65	47	5
COL1A2	*Bos taurus*	P02465	2131	53	45	8
COL1A2	*Ovis aries*	W5NTT7	1984	50	42	3
COL3A1	*Oryctolagus cuniculus*	G1T8J0	1221	32	98	7
COL3A1	*Bos taurus*	P04258	1123	28	26	3

Table S15: Collagen chains identified in the Fish glue 4 sample (HP6).

Gene Name	Taxonomy	Protein Identifier	Protein Score	Sequence Coverage (%)	No. of Peptides	No. of Unique Peptides

COL1A1	*Bos taurus*	P02453	2393	53	58	2
COL1A2	*Bos taurus*	P02465	2537	59	57	6
COL3A1	*Bos taurus*	P04258	843	31	25	6

Table S16: Collagen chains identified in the Fish glue 5 sample (HP7).

Gene Name	Taxonomy	Protein Identifier	Protein Score	Sequence Coverage (%)	No. of Peptides	No. of Unique Peptides
COL1A1	*Bos taurus*	P02453	2561	57	53	1
COL1A2	*Bos taurus*	P02465	2474	60	51	10
COL3A1	*Bos taurus*	P04258	1303	36	31	8

Table S17: Collagen chains identified in the Rabbit glue 6 sample (HM4).

Gene Name	Taxonomy	Protein Identifier	Protein Score	Sequence Coverage (%)	No. of Peptides	No. of Unique Peptides
COL1A2	*Bos taurus*	P02465	1463	46	31	5
COL1A1	*Bos taurus*	P02453	1430	41	34	0
COL1A2	*Oryctolagus cuniculus*	G1T2Z5	569	23	15	3
COL3A1	*Bos taurus*	P04258	485	16	11	2
COL3A1	*Oryctolagus cuniculus*	G1T8J0	251	8	6	1

Table S18: Collagen chains identified in the Rabbit glue 7 sample (HM5).

Gene Name	Taxonomy	Protein Identifier	Protein Score	Sequence Coverage (%)	No. of Peptides	No. of Unique Peptides
COL1A2	*Sus scrofa*	A0A1S7J1Y9	2617	61%	53	15
COL1A1	*Sus scrofa*	A0A287A1S6	2524	54%	52	7
COL3A1	*Sus scrofa*	F1RYI8	1949	43%	46	22
COL1A1	*Oryctolagus cuniculus*	G1T4A5	1757	48	38	1

Table S19: Collagen chains identified in the Strong Glue 8 sample (BM4).

Gene Name	Taxonomy	Protein Identifier	Protein Score	Sequence Coverage (%)	No. of Peptides	No. of Unique Peptides
COL1A1	*Bos taurus*	P02453	1845	65	45	1
COL1A2	*Bos taurus*	P02465	2048	63	48	9
COL1A1	*Equus asinus*	B9VR88	1624	57	40	4
COL1A2	*Equus asinus*	B9VR89	1290	48	31	7
COL1A2	*Sus scrofa*	A0A1S7J1Y9	1130	49	26	5

Table S20: Collagen chains identified in the Strong Glue 9 sample (BM5).

Gene name	Taxonomy	Protein Identifier	Protein Score	Sequence Coverage %	No. of Peptides	No. of Unique Peptides
COL1A1	*Bos taurus*	P02453	2337	53%	50	0
COL1A2	*Bos taurus*	P02465	2228	54%	48	7
COL1A1	*Sus scrofa*	A0A287A1S6	2242	50%	49	4
COL1A2	*Sus scrofa*	A0A1S7J1Y9	1860	48%	43	11
COL1A1	*Capra hircus* o *Ovis aries*	A0A452FHU9/ W5P481	2255	53%	49	0
COL1A2	*Ovis aries*	W5NTT7	1918	50%	42	2
COL1A1	*Equus asinus*	B9VR88	1930	44%	42	1
COL1A2	*Equus asinus*	B9VR89	1476	39%	33	5

Table S21: Collagen chains identified in the Rabbit Glue 10 sample (HP8).

Gene Name	Taxonomy	Protein Identifier	Protein Score	Sequence Coverage (%)	No. of Peptides	No. of Unique Peptides
COL1A1	*Bos taurus*	P02453	2498	56	52	1
COL1A2	*Bos taurus*	P02465	2512	57	51	9
COL3A1	*Bos taurus*	P04258	1654	39	38	9

Collagen modification: Backbone cleavage

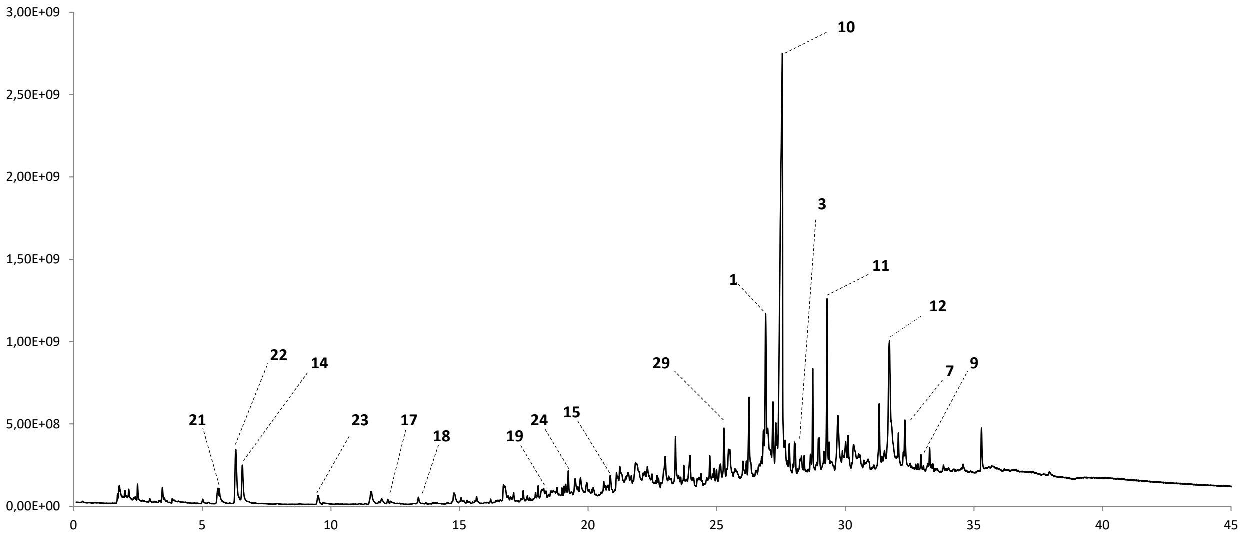

Figure S1. HP6 glue pyrogram. Peak numbering refers to compounds listed in Table S22

Table S22 Molecules identified in the pyrogram of HP6 and their most abundant mass spectrometric ions (*m/z*).

#	*Compound*	*Characteristic (fragment) ions (m/z)*
	DKPs	
**1**	**Cyclo(Pro-Ala)1**	**70, 97, 125, 168**
**2**	**Cyclo(Pro-Ala)2**	**70, 97, 125, 168**
**3**	**Cyclo(Pro-Val)1**	**70, 72, 125, 154, (196)**
**4**	**Cyclo(Pro-Val)2**	**70, 72, 125, 154, (196)**
**5**	**Cyclo(Pro-Met)1**	**70, 139, 154, 167, 228**
**6**	**Cyclo(Pro-Met)2**	**70, 139, 154, 167, 228**
**7**	**Cyclo(Pro-pyroGlu)**	**70, 96, 124, 152, 180, 208**
**8**	**Cyclo(Pro-Phe)1**	**70, 91, 125, 153, 244**
**9**	**Cyclo(Pro-Phe)2**	**70, 91, 125, 153, 245**
**10**	**Cyclo(Pro-Gly)**	**83, 98, 111, 154**
**11**	**Cyclo(Pro-Pro)**	**70, 96, 138, 166, 194**
**12**	**Cyclo(Pro-Hyp)1**	**70, 86, 124, 210**
**13**	**Cyclo(Pro-Hyp)2**	**70, 86, 124, 210**
	Aromatic hydrocarbons and heteroaromatics	
**14**	**Toluene**	**51, 65, 91**
**15**	**Benzenepropanenitrile**	**91, 92, 131**

16	Phenol	55, 66, 94
17	Ethyl-benzene	51, 65, 77, 91, 106
18	Styrene	51, 71, 104
19	Methyl-phenol	51, 77, 107
20	Methyl-pyrrole	53, 81
21	Pyridine	52, 79
22	Pyrrole	52, 67
23	Ethyl-pyrrole	53, 67, 80, 95
24	Benzeneacetonitrile	51, 63, 77, 90, 117
25	Diphenyl-ethylene	65, 91, 182
26	Propyl-benzene	65, 80, 91, 105, 120
27	Butenyl-benzene	65, 91, 104, 115, 132
28	Pyrazine	53, 8
29	Diethyl-pyrazine	53, 80, 107, 108, 136
30	2,5-Pyrrolidinedione	56, 99
	Fatty acids	
31	n-Hexadecanoic acid	60, 73, 129, 213, 256
32	9-Octadecenoic acid	69, 83, 97, 111, 264
33	Tetradecanoic acid	73, 97, 129, 185, 228
34	Octadecanoic acid	73, 129, 185, 241, 284

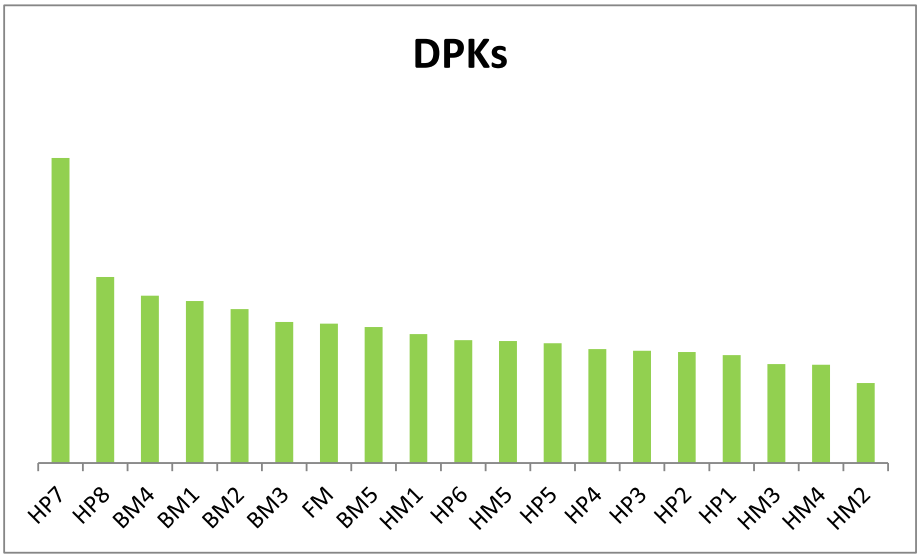

Figure S2. Sum of the areas of all DKPs detected normalized for the sample weight

Collagen damage: deamidation of glutamine and asparagine.

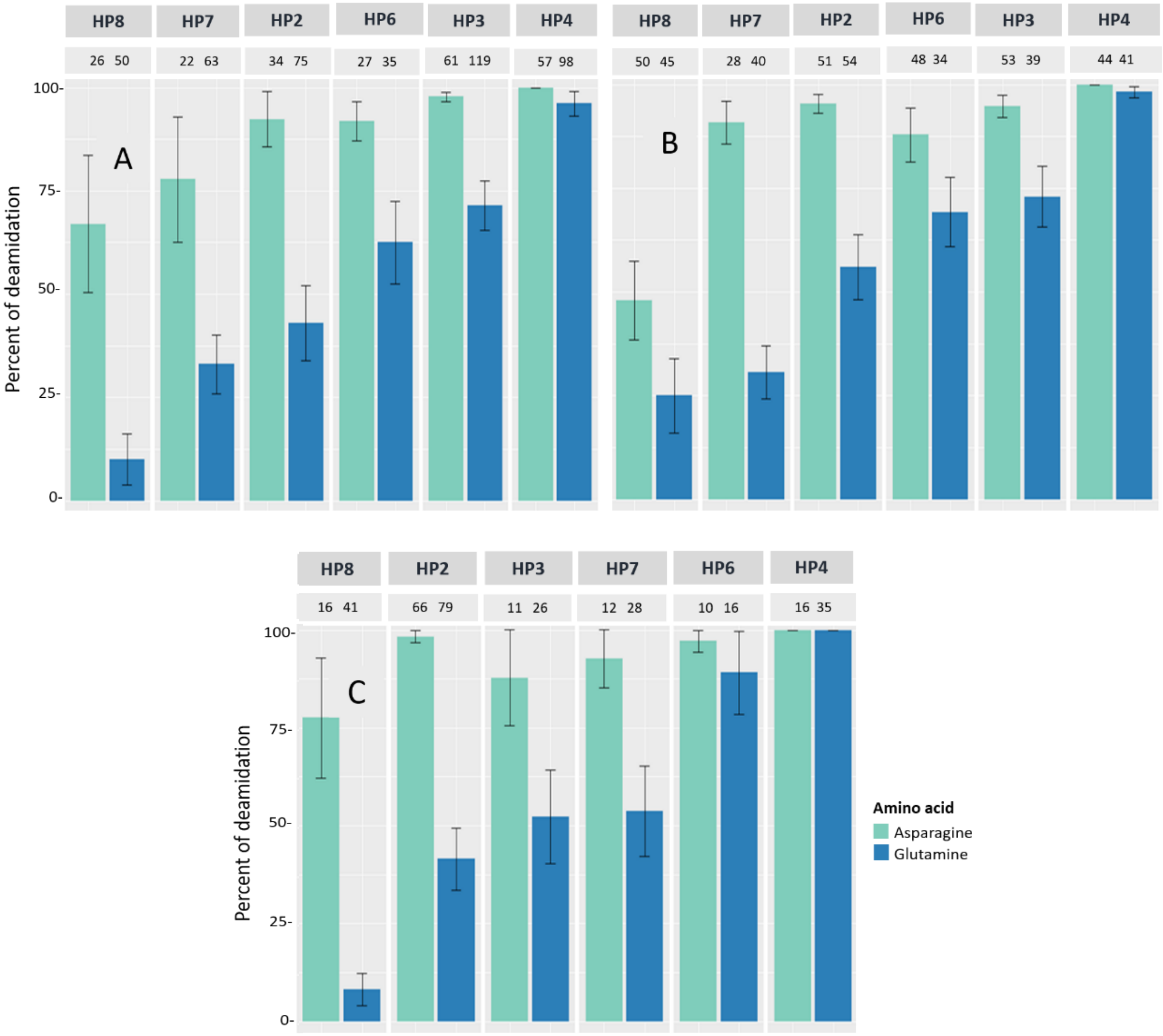

Figure S3: Overall percentage of deamidation of the single collagen chains in bovine pure hide glues (HP).

Semi-quantitative evaluation of deamidation for asparagines (N) and glutamines (Q) as calculated by MaxQuant software[1] with an in-house script based on peptide spectrum matches intensities[2], for collagen alpha-1(I) (A), collagen alpha-2(I) (B), and collagen alpha-1(III) (C). Error bars represent standard deviation and numbers above each bar represent the number of peptides the data is based on.

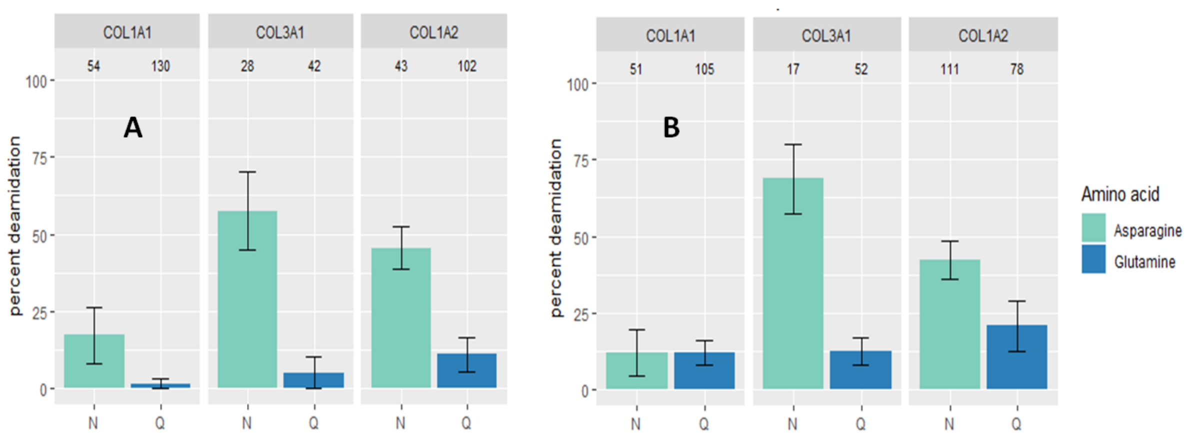

Figure S4: Overall percentage of deamidation of the single collagen chains in pure hide glues that contain only porcine glue (HP5) (A panel) and only rabbit glue (HP1) (B panel).

Semi-quantitative evaluation of deamidation for asparagines (N) and glutamines (Q) as calculated by MaxQuant software[1] with an in-house script based on peptide spectrum matches intensities[2] Error bars represent standard deviation and numbers above each bar represent the number of peptides the data is based on for the several chains of collagen

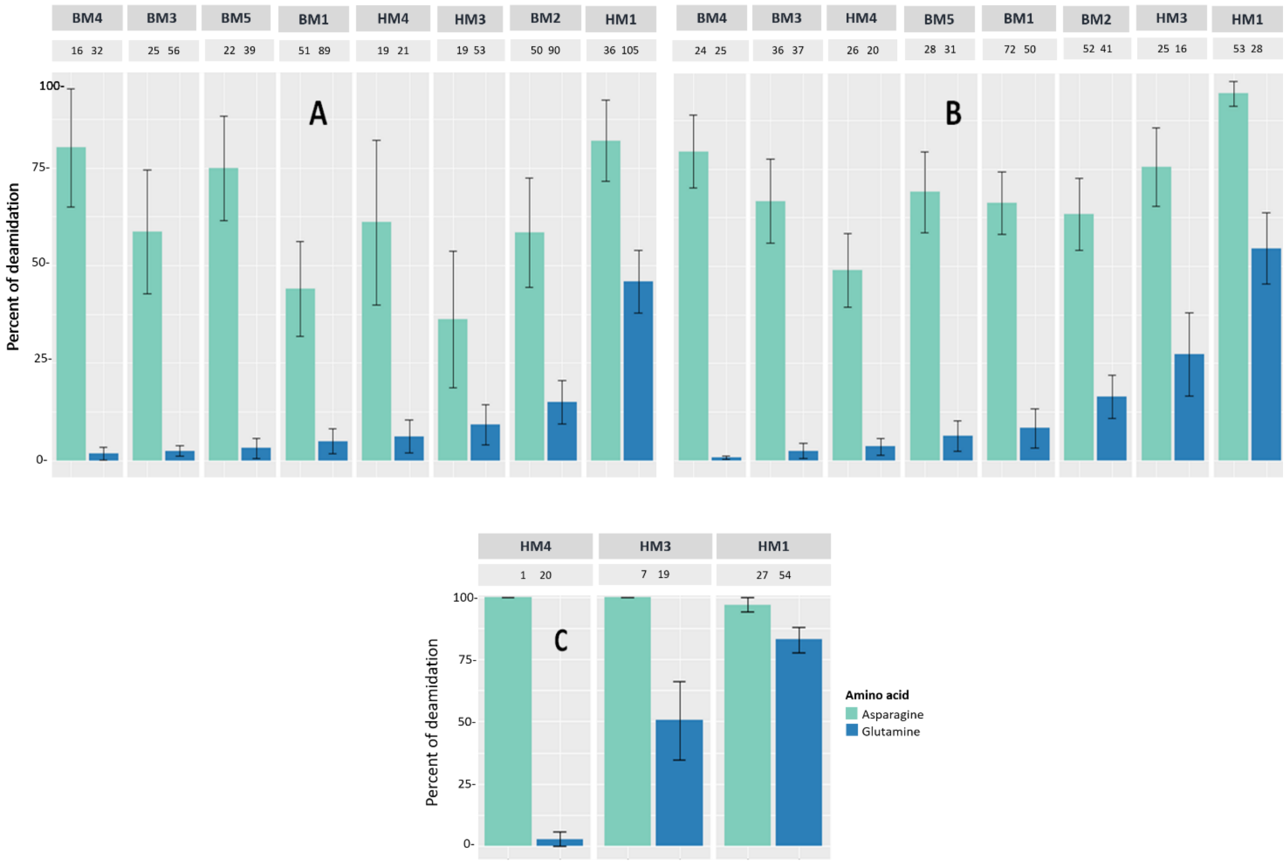

Figure S5: Overall percentage of deamidation of bovine collagen alpha-1(I) (A), collagen alpha-2(I) (B), and collagen alpha-1(III) (C) chains in mixed hide and bone glues containing bovine collagen.

Semi-quantitative evaluation of deamidation for asparagines (N) and glutamines (Q) as calculated by MaxQuant software[1] with an in-house script based on peptide spectrum matches intensities[2]. Error bars represent standard deviation and numbers above each bar represent the number of peptides the data is based on.

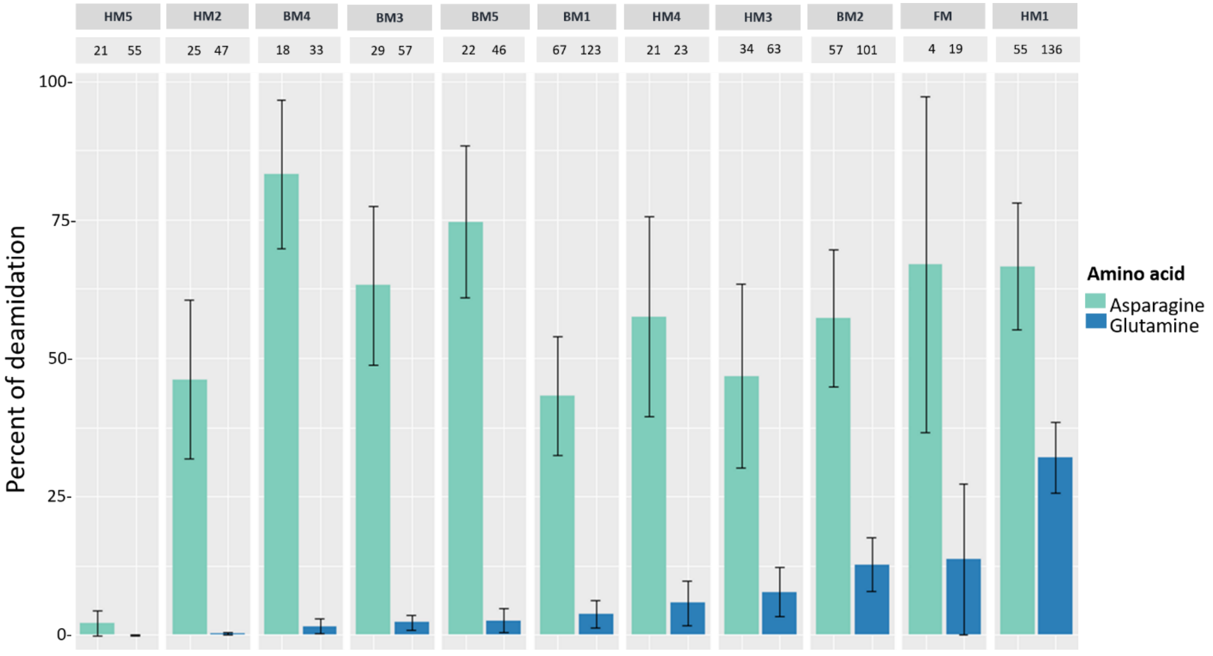

Figure S6: Overall percentage of deamidation of the protein family collagen alpha-1(I) in mixed animal glues.

Semi-quantitative evaluation of deamidation for asparagines (N) and glutamines (Q) was as calculated by MaxQuant software[1] with an in-house script based on peptide spectrum matches intensities[2]. Error bars represent standard deviation and numbers above each bar represent the number of peptides the data is based on.

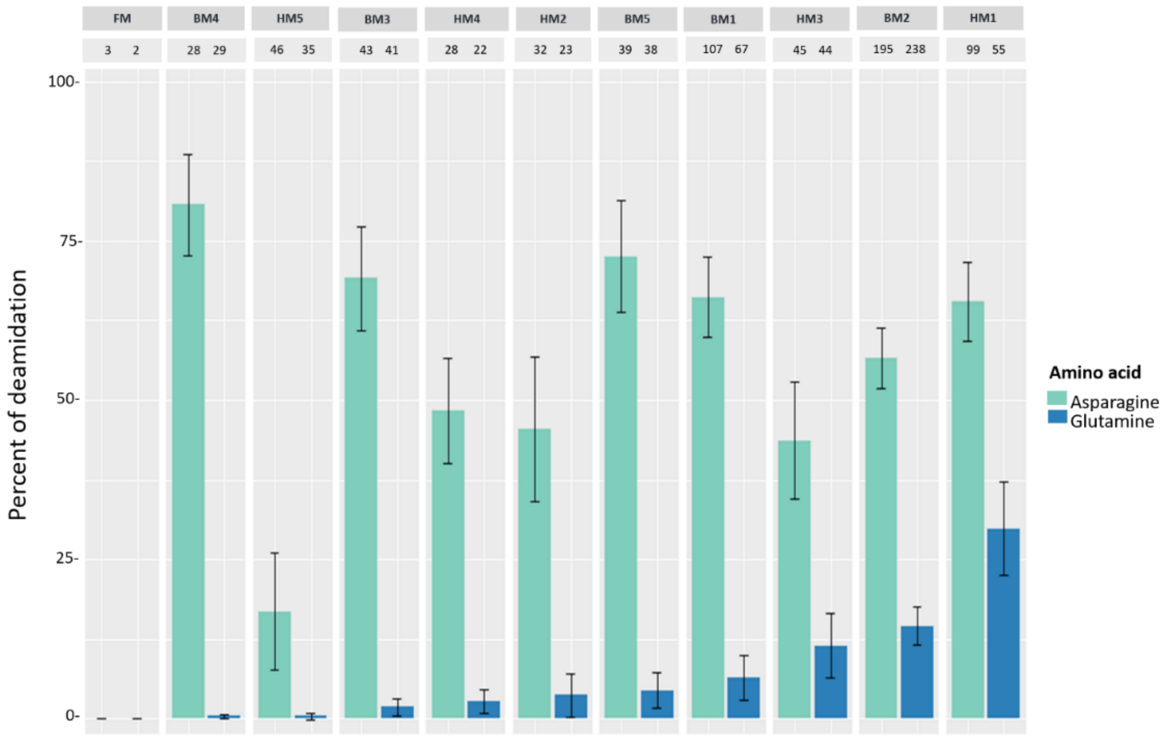

Figure S7: Overall percentage of deamidation of the protein family collagen alpha-2(I) in mixed animal glues.

Semi-quantitative evaluation of deamidation for asparagines (N) and glutamines (Q) was as calculated by MaxQuant software[1] with an in-house script based on peptide spectrum matches intensities[2]. Error bars represent standard deviation and numbers above each bar represent the number of peptides the data is based on.

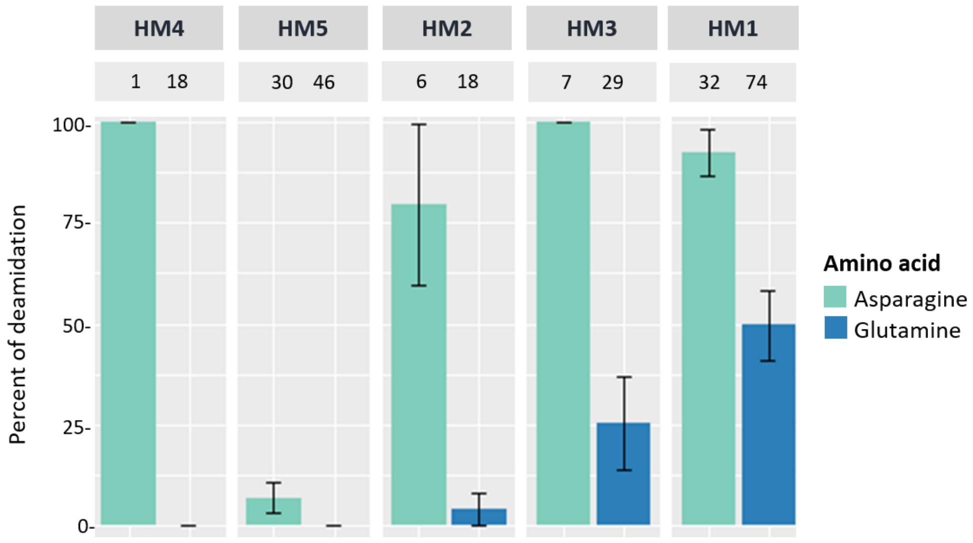

Figure S8: Overall percentage of deamidation of the protein family collagen alpha-1(III) in mixed animal glues. Semi-quantitative evaluation of deamidation for asparagines (N) and glutamines (Q) as calculated by MaxQuant software[1] with an in-house script based on peptide spectrum matches intensities[2]. Error bars represent standard deviation and numbers above each bar represent the number of peptides the data is based on.

Table S23: Site-specific evaluation of deamidation (N, Q) and oxidation (M) along *Bos taurus* collagen α1(I) sequence in all the pure bovine animal glues.

Asn and Gln detected as unmodified only (X); detected as deamidated (D); Detect both as unmodified and deamidated (DX); not detected at all (NF). Met detected as unmodified (X), as oxidized (O), both as unmodified and oxidized (OX); not detected at all (NF).

AA Position	HP3	HP4	HP2	HP6	HP7	HP8
162 Q	NF	NF	NF	NF	NF	NF
180 M	NF	NF	NF	NF	NF	NF
198 Q	X	X	DX	DX	DX	DX
201 Q	D	D	DX	DX	DX	DX
216 M	NF	NF	NF	NF	NF	NF
228 N	D	D	D	NF	NF	NF
249 Q	NF	NF	NF	NF	NF	NF
263 M	NF	NF	NF	NF	NF	NF
294 N	DX	D	D	DX	DX	NF
299 Q	DX	D	DX	DX	DX	D
300 M	OX	OX	O	O	O	O
323 N	DX	D	DX	DX	DX	DX
357 Q	DX	D	DX	DX	D	D
366 Q	D	D	D	DX	DX	D
386 N	DX	D	DX	DX	DX	DX
392 Q	DX	D	DX	DX	DX	DX
399 N	DX	D	DX	DX	DX	DX
420 Q	DX	D	DX	DX	DX	DX
431 N	D	D	D	NF	NF	NF
456 Q	DX	DX	DX	DX	DX	DX
560 Q	DX	D	DX	D	DX	DX
575 Q	DX	D	DX	D	DX	DX
579 M	OX	O	O	O	OX	O
618 Q	DX	D	DX	DX	DX	DX
633 Q	NF	NF	NF	NF	DX	NF
642 Q	NF	NF	NF	NF	DX	NF
660 Q	NF	NF	NF	NF	DX	NF
687 Q	DX	DX	DX	DX	DX	DX
699 N	DX	NF	DX	NF	NF	NF
704 N	DX	DX	DX	NF	NF	NF
720 Q	DX	D	DX	NF	DX	DX
726 Q	DX	X	DX	NF	DX	DX
728 M	OX	O	O	O	O	O
821 Q	DX	D	DX	DX	DX	DX
854 N	DX	D	DX	D	DX	DX
890 N	DX	D	DX	D	DX	DX
951 Q	DX	DX	DX	DX	DX	DX
956 Q	DX	D	DX	DX	DX	DX
965 Q	DX	D	DX	DX	DX	DX
984 Q	NF	NF	NF	NF	NF	NF
999 M	OX	OX	OX	O	OX	O

1089 Q	NF	NF	NF	NF	NF	NF
1101 Q	NF	NF	NF	NF	NF	NF
1116 Q	DX	DX	DX	DX	DX	DX
1128 Q	DX	DX	DX	DX	DX	DX
1155 N	DX	DX	DX	DX	DX	DX
1202 Q	NF	NF	NF	NF	NF	NF
1205 Q	NF	NF	NF	NF	NF	NF
1263 M	NF	NF	NF	NF	NF	NF
1292 M	NF	NF	NF	NF	NF	NF
1329 M	NF	NF	NF	NF	NF	NF
1357 M	NF	NF	NF	NF	NF	NF
1376 M	NF	NF	NF	NF	NF	NF

Table S24: Site-specific evaluation of deamidation (N, Q) and oxidation (M) along *Bos taurus* collagen α2(I) sequence in all the pure bovine animal glues.

Asn and Gln detected as unmodified only (X); detected as deamidated (D); Detect both as unmodified and deamidated (DX); not detected at all (NF). Met detected as unmodified (X), as oxidized (O), both as unmodified and oxidized (OX); not detected at all (NF).

AA Position	HP3	HP4	HP2	HP6	HP7	HP8
80 Q	NF	NF	NF	NF	NF	NF
91 M	NF	NF	NF	NF	NF	NF
94 M	NF	NF	NF	NF	NF	NF
109 Q	NF	NF	NF	DX	DX	DX
112 Q	NF	NF	X	DX	DX	DX
123 Q	NF	DX	DX	DX	DX	DX
160 Q	NF	DX	NF	D	DX	DX
181 N	NF	NF	NF	NF	NF	DX
189 Q	D	NF	DX	NF	NF	NF
205 N	D	DX	DX	NF	NF	NF
210 Q	NF	DX	DX	NF	NF	NF
270 N	DX	NF	X	D	D	NF
297 N	DX	DX	DX	DX	DX	DX
301 N	D	DX	DX	DX	DX	DX
357 N	NF	NF	NF	NF	DX	DX
366 Q	NF	NF	DX	NF	DX	DX
399 N	NF	NF	NF	NF	NF	NF
415 M	NF	NF	NF	O	X	O
433 N	DX	DX	DX	D	DX	DX
445 M	OX	OX	OX	O	X	OX
456 N	D	DX	DX	D	DX	DX
489 N	D	DX	X	DX	DX	DX
525 N	NF	NF	DX	DX	DX	DX
526 N	NF	NF	DX	DX	DX	DX
529 Q	NF	NF	DX	DX	DX	DX
535 Q	NF	DX	X	DX	DX	X

538 Q	NF	NF	DX	DX	DX	X
544 Q	D	NF	X	DX	DX	DX
553 Q	NF	NF	DX	D	DX	DX
618 N	D	DX	DX	D	DX	DX
688 N	D	DX	DX	D	DX	DX
721 N	D	DX	DX	D	D	DX
732 Q	DX	DX	DX	D	DX	DX
748 N	D	DX	DX	D	DX	DX
766 N	D	DX	DX	DX	DX	DX
823 Q	NF	DX	NF	NF	NF	NF
862 Q	NF	DX	DX	DX	DX	DX
909 N	D	DX	DX	DX	DX	DX
912 N	DX	DX	DX	DX	DX	DX
916 N	D	DX	DX	DX	D	DX
927 N	D	NF	DX	DX	NF	NF
930 N	NF	NF	NF	NF	NF	NF
939 Q	NF	NF	NF	NF	NF	NF
951 N	DX	NF	NF	NF	NF	DX
964 Q	DX	DX	DX	DX	DX	DX
975 N	NF	DX	DX	D	D	NF
1000 Q	NF	DX	NF	NF	DX	NF
1024 N	D	NF	DX	DX	DX	DX
1027 Q	D	DX	DX	DX	DX	DX
1039 Q	DX	NF	DX	DX	DX	DX
1068 Q	DX	DX	DX	DX	DX	DX
1081 Q	NF	NF	NF	NF	NF	NF
1084 Q	NF	NF	NF	NF	NF	NF
1186 M	NF	NF	NF	NF	NF	NF
1248 M	NF	NF	NF	NF	NF	NF
1255 M	NF	NF	NF	NF	NF	NF
1277 M	NF	NF	NF	NF	NF	NF

Table S25. Accession entries as in the COLLE database and their correspondence in Uniprot database

Entry as in "colle database"	Protein	Gene	Organism	Uniprot accession number
AAI05185.1	Collagen type I alpha 1	COL1A1	*Bos taurus*	P02453
AAI49096.1	Collagen type I alpha 2	COL1A2	*Bos taurus*	P02465
AAI23470.1	Collagen type IIII alpha 1	COL3A1	*Bos taurus*	Q08E14
A0A287A1S6	Collagen type I alpha 1	COL1A1	*Sus scrofa*	A0A287A1S6
NP_001230584.1	Collagen type I alpha 2	COL1A2	*Sus scrofa*	A0A1S7J1Y9
NP_001230226.1	Collagen type III alpha 1	COL3A1	*Sus scrofa*	F1RYI8
G1T4A5	Collagen type I alpha 1	COL1A1	*Oryctolagus cuniculus*	G1T4A5
NP_001182597.1	Collagen type I alpha 2	COL1A2	*Oryctolagus cuniculus*	G1T2Z5
G1T8J0	Collagen type III alpha 1	COL3A1	*Oryctolagus cuniculus*	G1T8J0
W5P481	Collagen type I alpha 1	COL1A1	*Ovis aries*	W5P481
XP_004007775.1	Collagen type I alpha 2	COL1A2	*Ovis aries*	W5NTT7
W5Q4S0	Collagen type III alpha 1	COL3A1	*Ovis aries*	W5Q4S0
COL1A1-201	Collagen type I alpha 1	COL1A1	*Capra hircus*	A0A452FHU9
XP_005678993.1	Collagen type I alpha 2	COL1A2	*Capra hircus*	A0A452G3V6
XP_005675926.1	Collagen type III alpha 1	COL3A1	*Capra hircus*	N/A
AAH50014.1	Collagen type I alpha 1	COL1A1	*Mus musculus*	P11087
AAH42503.2	Collagen type I alpha 2	COL1A2	*Mus musculus*	Q01149
AAH52398.1	Collagen type III alpha 1	COL3A1	*Mus musculus*	Q7TT32
P02454	Collagen type I alpha 1	COL1A1	*Rattus norvegicus*	P02454
NP_445808.1	Collagen type I alpha 2	COL1A2	*Rattus norvegicus*	P02466
AAH87039.1	Collagen type III alpha 1	COL3A1	*Rattus norvegicus*	P13941
NP_001310708.1	Collagen type I alpha 1	COL1A1	*Equus asinus*	B9VR88
NP_001310709.1	Collagen type I alpha 2	COL1A2	*Equus asinus*	B9VR89
F7BJV7	Collagen type I alpha 1	COL1A1	*Macaca mulatta*	F7BJV7
NP_001253266.1	Collagen type I alpha 2	COL1A2	*Macaca mulatta*	H9Z2D1
NP_001003090.1	Collagen type I alpha 1	COL1A1	*Canis lupus familiaris*	Q9XSJ7
NP_001003187.1	Collagen type I alpha 2	COL1A2	*Canis lupus familiaris*	O46392
M3W2F5	Collagen type I alpha 1	COL1A1	*Felix catus*	M3W2F5
AAH63249.1	Collagen type I alpha 1	COL1A1	*Danio rerio*	Q6P4U1
AAH71278.1	Collagen type I alpha 2	COL1A2	*Danio rerio*	Q6IQX2
P02457	Collagen type I alpha 1	COL1A1	*Gallus gallus*	P02457
NP_001073182.2	Collagen type I alpha 2	COL1A2	*Gallus gallus*	A0A5H1ZRJ7
P12105	Collagen type III alpha 1	COL3A1	*Gallus gallus*	P12105
NP_001116390.2	Collagen type I alpha 1 precursor	COL1A1	*Oryzias latipes*	A8QX86
H2M6N2	Collagen type I alpha 1b	COL1A1	*Oryzias latipes*	H2M6N2
NP_001080821.1	Collagen type I alpha 1 S homeolog precursor	COL1A1	*Xenopus laevis*	Q802B5
NP_001080727.1	Collagen type I alpha 2 L homeolog precursor	COL1A2	*Xenopus laevis*	Q801M5
SBS49270.1	Collagen type I alpha 2	COL1A2	*Nothobranchius Furzeri*	A0A1A8UMG6
NP_001117679.1	Collagen type I alpha 2	COL1A2	*Oncorhynchus mykiss*	O93484
BAL40987.1	Collagen type I alpha 1	COL1A1	*Oreochromis niloticus*	G9M6I5

NP_001269826.1	Collagen type I alpha 2	COL1A2	*Oreochromis niloticus*	G9M6I6
BAG72200.1	collagen type I alpha 1	COL1A1	*Carassius auratus*	B5U968
BAG72201.1	Collagen type I alpha 2	COL1A2	*Carassius auratus*	B5U969
ADK35755.1	collagen type I alpha 1	COL1A1	*Ctenopharyngodon idella*	E2GK07
ADK48763.1	Collagen type I alpha 2	COL1A2	*Ctenopharyngodon idella*	E2IPR2
ABY71227.1	collagen type I alpha 1 partial	COL1A1	*Scyliorhinus canicula*	D0PQF7
ABY71228.1	Collagen type I alpha 2 partial	COL1A2	*Scyliorhinus canicula*	D0PQF8
XP_013991798.1	collagen type I alpha 1	COL1A1	*Salmo salar*	A0A1S3LLE2
XP_014072147.1	Collagen type I alpha 2 isoform X1	COL1A2	*Salmo salar*	A0A1S3T6D0
XP_014072148.1	Collagen type III alpha 1 Isoform X2	COL3A1	*Salmo salar*	A0A1S3T6D7
U3IW58	Collagen type I alpha 2	COL1A2	*Anas platyrhynchos*	U3IW58
G1NB83	Collagen type I alpha 2	COL1A2	*Meleagris gallopavo*	G1NB83 (deleted)
W5N610	collagen type I alpha 1b	COL1A1	*Lepisosteus oculatus*	W5N610
W5MY87	Collagen type I alpha 2	COL1A2	*Lepisosteus oculatus*	W5MY87
W5MGF7	Collagen type III alpha 1	COL3A1	*Lepisosteus oculatus*	W5MGF7
W5L8R7	collagen type I alpha 1a	COL1A1	*Astyanax mexicanus*	W5L8R7
W5K5Z0	collagen type I alpha 1b	COL1A1	*Astyanax mexicanus*	W5K5Z0
W5LPK3	Collagen type I alpha 2	COL1A2	*Astyanax mexicanus*	W5LPK3
H2UF52	collagen type I alpha 1a	COL1A1	*Takifugu rubripes*	H2UF52 (deleted)
H2T1T7	collagen type I alpha 1b	COL1A1	*Takifugu rubripes*	H2T1T7 (deleted)
H2UR13	Collagen type I alpha 2	COL1A2	*Takifugu rubripes*	H2UR13 (deleted)

References

(1) Cox, J.; Mann, M. MaxQuant Enables High Peptide Identification Rates, Individualized p.p.b.-Range Mass Accuracies and Proteome-Wide Protein Quantification. *Nat. Biotechnol.* **2008**, *26* (12), 1367–1372.

(2) Mackie, M.; Rüther, P.; Samodova, D.; Di Gianvincenzo, F.; Granzotto, C.; Lyon, D.; Peggie, D. A.; Howard, H.; Harrison, L.; Jensen, L. J.; Olsen, J. V.; Cappellini, E. Palaeoproteomic Profiling of Conservation Layers on a 14th Century Italian Wall Painting. *Angew. Chemie - Int. Ed.* **2018**, *57* (25), 7369–7374.
